# Vestibular Disability/Handicap in Fibromyalgia: A Questionnaire Study

**DOI:** 10.3390/jcm11144017

**Published:** 2022-07-11

**Authors:** Viviana Mucci, Ilaria Demori, Fabio Rapallo, Elena Molinari, Serena Losacco, Lucio Marinelli, Cherylea J. Browne, Bruno Burlando

**Affiliations:** 1 School of Science, Western Sydney University, Sydney, NSW 2751, Australia or v.mucci@westernsydney.edu.au (V.M.); c.browne@westernsydney.edu.au (C.J.B.); 2 Department of Earth, Environmental and Life Sciences (DISTAV), University of Genova, Corso Europa, 26, 16132 Genova, Italy; 3 Department of Economics (DIEC), University of Genova, Via Vivaldi, 5, 16126 Genova, Italy; fabio.rapallo@unige.it; 4 Clinical Psychology Unit, E.O. Ospedali Galliera, Via Mura delle Cappuccine 14, 16128 Genova, Italy; elena.molinari@galliera.it; 5 Department of Pharmacy, DIFAR, University of Genova, Viale Benedetto XV, 3, 16132 Genova, Italy; losacco@difar.unige.it (S.L.); bruno.pietro.burlando@unige.it (B.B.); 6 Department of Neuroscience, Rehabilitation, Ophthalmology, Genetics, Maternal and Child Health, DINOGMI, University of Genova, Largo P. Daneo 3, 16132 Genova, Italy; lucio.marinelli@unige.it; 7 IRCCS Ospedale Policlinico San Martino, Department of Neuroscience, Division of Clinical Neurophysiology, Largo R. Benzi 10, 16132 Genova, Italy

**Keywords:** Cognitive Behavioural Assessment-Hospital, Dizziness Handicap Inventory, painDETECT questionnaire, persistent postural-perceptual dizziness, Situational Vertigo Questionnaire, visually induced dizziness

## Abstract

Fibromyalgia (FM) is a poorly understood, central pain processing disorder characterized by a broad range of symptoms, such as chronic pain, sleep disruption, chronic fatigue, and psychosomatic symptoms. In addition, recent studies have shown that FM patients also experience dizziness. We aimed to establish a prevalence rate of vestibular symptoms in a population of FM patients through a battery of questionnaires investigating socio-demographic, clinical and psychological characteristics, combined with the Dizziness Handicap Inventory (DHI) and the Situational Vertigo Questionnaire (SVQ). A total of 277 respondents, officially diagnosed with FM, completed the full study, while 80 controls were also included for DHI and SVQ questionnaires. We found that FM participants were significantly affected by vestibular symptoms, which correlated with FM-associated pain and non-pain symptoms. The dizziness reported by FM participants showed peculiar features suggesting an FM-intrinsic mechanism of vestibular dysfunction, possibly linked to migraine and dysautonomia conditions. Correlations between dizziness and depressive mood (or neuroticism), revealed an impact of dizziness on psychological status, leading to depressive reactions and interpersonal difficulties, and possibly involving a noxious, self-sustained stress condition. In conclusion, data showed a manifesting dizziness condition in FM patients that warrants careful clinical attention due to its possible inherent role in the syndrome.

## 1. Introduction

Fibromyalgia (FM) is a central pain processing disorder characterized by idiopathic, chronic, and wide-spread pain. However, accumulating evidence indicates that FM is not a stand-alone diagnosis, as in most cases it is associated with a wide range of symptoms and several comorbidities, including sleep disorders, chronic fatigue, mood disorders, cognitive dysfunctions, headache, irritable bowel syndrome and migraine. These disorders have been classified as a “central sensitivity syndrome” (CSS) but their association and how their pathophysiology might be interrelated remains unknown [1,2]. Besides the understanding that FM involves disordered central pain processing, another innovative view consists of its characterization as a multi-sensory syndrome, i.e., arising from more than one of the sensory systems, or a dysfunction in sensory integration [3]. FM patients also report vertigo and dizziness, described as instability [4,5,6,7]. A recent study concluded that the feelings of dizziness and unsteadiness of the patients with FM might be amplified by CSS [6]. In addition to dizziness, patients are also reporting neck pain and headaches [4,6,8]. It remains debatable if headache and neck pain are part of FM per se or associated with vertigo and the dizziness component of FM, given that many vestibular patients often report similar complains [9,10]. Moreover, visual snow syndrome, which is associated with “perceptual disorders”, has been reported in FM patients, together with tinnitus and dizziness [11]. Hence, understanding whether these symptoms are a psychosomatic component, or are derived from peripheral or central comorbidities, is vital for managing these patients and improving their care.

Even though the clinical characterization of dizziness in FM patients is still poor, a clue of its prevalence in the condition derives from the correlation between FM and migraine. The prevalence of migraine in FM has been established [12,13,14,15] and in addition, migraine has also been shown to affect vestibular-related disorders (e.g., vestibular migraine, chronic subjective dizziness, etc.) [16], suggesting the involvement of a common central processing dysfunction. This further supports the assessment of the vestibular component in FM pathophysiology.

The prevalence of vestibular symptoms in FM patients and the presence of FM in vestibular patients remain largely unexplored. In 2011, a study evaluated the vestibular functions of FM patients through peripheral function tests, focusing solely on potential peripheral vestibular disorders [7]. This paper was one of the few to analyze and assess the vestibular involvement in FM patients from empirical evidence, overall suggesting that vestibular dysfunction in FM patients should be further explored.

Given these considerations, the present study aimed to establish a prevalence rate of vestibular symptoms in FM, including handicap provoked by dizziness and correlations between vestibular symptoms and cognitive/behavioral characteristics and mood. The first aim of this study was to assess if the prevalence of vestibular problems is higher in FM respondents with respect to the general population and how dizziness affects FM patients. For this, a control group of participants was enrolled in the study and patients and controls were asked to complete a Dizziness Handicap Inventory (DHI) and Situational Vertigo Questionnaire (SVQ). The DHI is one of the most commonly used questionnaires in vestibular medicine and it was developed to evaluate the self-perceived handicapping effects imposed by vestibular system diseases [17]. The SVQ is a questionnaire specifically designed to identify the presence of visual vertigo, also named visual vestibular mismatch or visually induced motion sickness, and currently named visually induced dizziness (VID). The latter is a condition attributable to a defective vestibular compensation strategy, where the subject in order to compensate for a vestibular deficit (either peripheral or central) becomes overly dependent on the available visual information [18,19]. VID is common in patients affected by vestibular migraine and migraine [20] and is considered part of a central component of dizziness [21].

Following this first screening, FM participants were also asked about pain and non-pain symptoms, to be put in correlation with vestibular symptoms, through a series of questionnaires. In addition, stress and psychological components were also considered, given that dizziness might either depend on vestibular problems or on anxiety and stress. Moreover, it is known that psychological factors and stressful events, particularly trauma, abuse and neglect experienced in early life, play a role in FM pathogenesis and pathophysiology [22]. Early-life stressors can impair the programming of the stress responses, thus increasing vulnerability to stressors in adult life [23]. Accordingly, FM patients are characterized by psychological disorders including depression, anxiety, and post-traumatic stress [24], which generally result in experiencing more intense pain [25], as well as dizziness and loss of balance, which are common neurovegetative symptoms related to high levels of stress and anxiety [26]. Hence, we also explored the relationship between cognitive/behavioral characteristics of FM participants, their early-life stressful experiences, and the presence of vestibular symptoms.

## 2. Materials and Methods

### 2.1. Ethical Approval, Study Population and Recruitment

The recruitment and study of FM respondents was approved by the Research Ethics Committee of the University of Genova (Assent N. 2021/32). Each respondent provided informed consent. All investigations were conducted according to the principles expressed in the Declaration of Helsinki. Participants with an established FM diagnosis were recruited by an Italian association of people affected by FM (Fibromialgia Comitato Assoutenti Liguria, https://www.facebook.com/FibromialgiaComitatoAssoutentiLiguria/ (accessed on 1 April 2021), https://comitatofibromialgia.blogspot.com (accessed on 1 April 2021)). Only patients who were officially diagnosed by an accredited healthcare professional were included in the FM group. We considered this as validated diagnosis and formed part of the primary inclusion criteria. Following this, the subjects were asked to complete an extensive pre-enrollment questionnaire, which was then reviewed by our team of physicians and healthcare professionals. If there was any ambiguity regarding time of onset or symptoms reported, they were excluded from the study. Multiple exclusion criteria were adopted, i.e., unfamiliarity with the Italian language, pregnancy or breastfeeding, abuse of substances or alcohol, and psychiatric disorders.

Data from control subjects were collected in a previous vestibular study led by VM and CB, conducted in 2019–2022. The Western Sydney University Human Ethics Committee provided ethical approval (H11962). A total of 80 subjects recruited online from all over the world, out of the 197 control respondents, provided informed consent for the researchers to also use their deidentified data in the current study. All investigations were conducted according to the principles expressed in the Declaration of Helsinki.

### 2.2. Questionnaires

Data from FM patients were collected via an online anonymous survey from April to June 2021 (Microsoft Forms ^®^, https://www.office.com (accessed on 6 February 2021), Microsoft, Redmond, WA, USA). The first section of the survey on FM patients assessed socio-demographic characteristics such as age, gender, family status, educational status, working status, and in addition, clinical aspects including biometric parameters, age-of-onset, diagnostic delay, and disease duration. Symptoms other than pain, including among others, fatigue, sleep disturbance, and migraine, were recorded on a presence/absence basis.

Pain evaluation was achieved through a survey on global pain intensity by using: (i) the numerical pain rating scale (scale 0–10; 0 = “no pain”; 10 = “worst imaginable pain”) [27], (ii) a series of pain types (scale 1–5, pressure pain, numbness, tingling, sudden pain, burning, light contact, occasional pain), and (iii) an Italian version of the painDETECT questionnaire (PD-Q) for the classification of pain perception into nociceptive or neuropathic/central pain [28]. A PD-Q score ≤ 12 indicates that a neuropathic pain component is unlikely, a PD-Q score ≥ 19 indicates that a neuropathic or central pain component is >90% likely, and an intermediate condition is considered in between.

FM participants were asked to rate their childhood and adolescence on a scale from 1 to 5 (very bad, bad, average, good, excellent) and to state what traumatic events occurred in their life and in which year.

For the evaluation of cognitive and behavioral management, participants affected by FM answered an Italian version of the Cognitive Behavioural Assessment-Hospital (CBA-H) [29]. The CBA-H consists of broad-spectrum, “true/false” questions, aiming at multiple evaluations including anxiety, well-being, depression, psychological distress, fear, and stable personality traits.

For the evaluation of vestibular problems, FM patients answered validated Italian versions of the Dizziness Handicap Inventory (DHI) [30] and the Situational Vertigo Questionnaire (SVQ) [31]. The DHI consists of 25 items with yes/no/sometimes questions, corresponding to 0, 2, and 4 scores, respectively. Based on total scores, four cutoffs are defined, corresponding to: ≤14: no handicap, 16–34: mild handicap, 36–52: moderate handicap, and ≥54: severe handicap. In addition, the DHI items are sub-grouped into three domains representing Functional, Emotional, and Physical components of dizziness. The Physical component poses questions regarding certain movements that could increase the patient’s symptoms, (e.g., “Does looking up increases your problem?”; “Do quick movements of your head increase your problem?”). The Emotional component focuses on how the patient is perceived (e.g., “Because of your problem, do you feel frustrated?”; “Because of your problem, have you been embarrassed in front of others?”). The Functional component evaluates the interference of dizziness on the performance of certain eye, head, and body movements, focusing on the capacity of performing various activities and tasks such as walking without help and in the dark (e.g., “Because of your problem, do you have difficulty reading?”; “Because of your problem, do you avoid heights?”). The SVQ consists of 19 items aiming at recognizing visual vertigo, with a score ranging from 0 (not at all) to 4 (very much) for each item.

For control subjects, a 49-item questionnaire was distributed using the online survey platform Qualtrics (https://www.qualtrics.com/qualtrics/xm (accessed on 1 February 2021), Seattle, WA, USA). Only data relating to gender, age, and vestibular aspects (English versions of the DHI and SVQ questionnaires) were analyzed in this study.

### 2.3. Statistical Analyses

Sociodemographic and clinical data were analyzed by descriptive statistics. Correlation analyses were conducted using the Spearman’s rank correlation coefficient, while for inference analyses, the Pearson’s chi-squared and Wilcoxon rank sum tests were used. The multivariate analysis of the DHI and SVQ questionnaires was carried out through cluster analysis with Manhattan distance and complete linkage aggregation, and through factor analysis. The number of factors in exploratory factor analysis was determined with the optimal coordinate criterion, while confirmatory factor analysis was based on the Tucker-Lewis Index. All data analyses were carried out using the software R (version 4.0.5, https://www.r-project.org/ (accessed on 6 April 2022)).

## 3. Results

### 3.1. Clinical Characteristics

**Figure 1:**
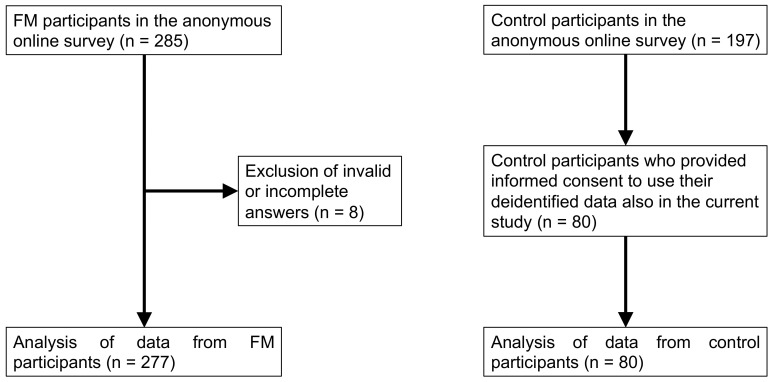
Workflow of participant inclusion/exclusion criteria and process applied in the study.

The survey received 285 answers from FM participants, of which 277 were validated and used in the present study (details reported in Figure 1). FM patients were prevalently females with upper secondary, or higher, education, married or cohabitant, and with white-collar employment (Table 1), while clinical features showed the typical pattern of the disease (Table 2). Most FM applicants (67.5%) were diagnosed by a rheumatologist, while only 3.2% by a neurologist, and the remaining ones by other medical specialists. In addition, only 5.6% of patients were diagnosed for vestibular problems by a qualified specialist.

Given that FM is characterized by a complex of symptoms, FM participants were also asked to report symptoms other than pain. The sum of these data was higher than the number of participants since, as expected, many of them reported more than one symptom. The percent occurrence of each non-pain symptom in FM patients is reported in Table 3. Dizziness was here referred in general terms as instability, disequilibrium and vertigo [32]; no clear distinction among spinning rotatory vertigo and general disequilibrium was given for this question related to dizziness as a non-pain symptom in FM participants.

### 3.2. Comparisons between FM Patients and Controls

When considering the FM population versus controls (277 FM participants vs. 80 control participants, see Materials and Methods and Figure 1), female prevalence was higher in the FM group (Table 1), but the age distribution was not significantly different from the FM group (Table 2). The statistics of DHI overall scores and SVQ scores (Table 4) showed a significantly higher incidence of vestibular symptoms reported by FM participants with respect to controls, as confirmed by the Wilcoxon rank sum test (Table 4). The distribution of subjects in the different handicap levels was 5.8% no handicap, 15.5% mild handicap, 28.5% moderate handicap, and 50.2% severe handicap, for FM patients, while it was 88.8% no handicap, 5% mild handicap, 2.5% moderate handicap, and 2.5% severe handicap, for control subjects.

### 3.3. Correlations between Vestibular Scores and Pain Perception

These analyses and the following ones were conducted on the data from FM participants only. The PD-Q test scores had a median of 22, with an interquartile range of 17–27, and a mean value of 21.48. These statistics indicate a sharp prevalence of neuropathic or central pain. The correlation between DHI overall scores and PD-Q test scores, measured by the Spearman’s rank correlation coefficient, yielded a value of ρ_s_ = 0.443, while the correlation between DHI overall scores and pain intensity in the last week was ρ_s_ = 0.346. As for the SVQ scores, their correlation with the PD-Q test and pain intensity in the last week were ρ_s_ = 0.349 and ρ_s_ = 0.253, respectively. Spearman’s correlation analysis between the FM pain types, as defined in the questionnaire, and the scores of the DHI and SVQ questionnaires, yielded ρ_s_ values ranging between 0.264 and 0.395 for the DHI test and between 0.217 and 0.319 for the SVQ test (Table 5). These correlations indicate that DHI severe handicap and higher visual dizziness tend to be associated with neuropathic or central pain (higher PD-Q scores) and higher pain intensity.

### 3.4. Correlations between DHI Handicap Levels and Diagnosis Delay or PD-Q Scores

Based on the DHI test cutoffs, a dizziness handicap level can be assigned to each of the 277 FM participants, resulting in 16 subjects rating as “no handicap”, 43 “mild handicap”, 79 “moderate handicap”, and 139 “severe handicaps”. These data are significantly different from a random distribution of subjects among the four handicap levels (chi-squared test, *p* < 0.001), revealing a tendency for high dizziness handicap in FM participants. DHI handicap levels were also correlated with diagnosis delay and PD-Q scores, with ρ_s_ = 0.146 and 0.442, respectively.

### 3.5. Associations between Vestibular Tests and CBA-H or Non-Pain Symptoms

**Figure 2:**
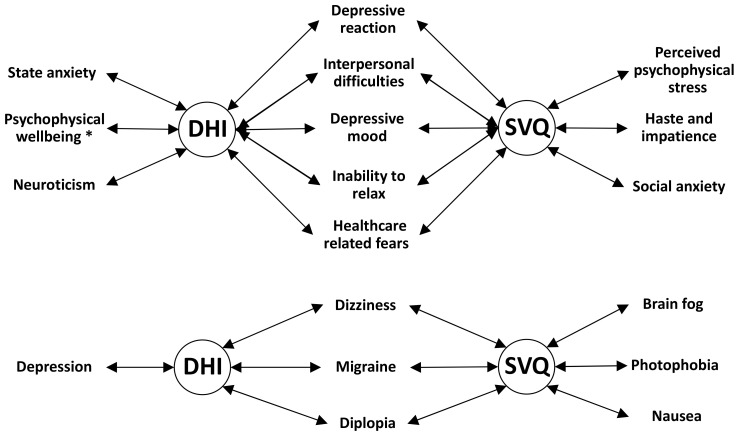
Significant associations between Dizziness Handicap Inventory (DHI) or Situational Vertigo Questionnaire (SVQ) scores and the indicated CBA-H items (above) or non-pain symptoms (below). DHI: significant associations are highlighted by the analysis of contingency tables of the DHI handicap levels cross-classified by the CBA-H cutoffs, or cross-classified by the presence/absence of symptoms (*p* < 0.01, chi-squared test). SVQ: significant associations are indicated by the SVQ scores cross-classified by the CBA-H cutoffs, or cross-classified by the presence/absence of symptoms (*p* < 0.01, Wilcoxon rank sum test). In all cases, higher DHI or SVQ scores correspond to presence of condition or symptoms, except Psychophysical wellbeing (*) for which higher DHI scores correspond to lack of condition.

Various significant associations were found between the DHI overall scores and the results of the CBA-H questionnaire, as shown by chi-squared analysis applied to contingency tables of the dizziness handicap data cross-classified by the clinical cutoffs of CBA-H scales, which indicate the presence or absence of different cognitive/behavioral conditions (Figure 2). A similar analysis revealed associations between dizziness handicap and the presence or absence of various symptoms other than pain as reported by FM participants (Figure 2). It should be noted that the symptoms “fatigue” and “sleep disturbance” were not included in this analysis because they were reported by almost all subjects.

Significant associations were also found between the SVQ on one side and the CBA-H questionnaire, or symptoms other than pain, on the other side. In this case, the Wilcoxon rank sum test was applied to the SVQ scores cross-classified by the clinical cutoffs of each CBA-H scale, or by the presence or absence of each symptom (Figure 2). In all cases, significant associations indicated the presence of higher DHI and SVQ scores in concomitance with subclinical/clinical conditions for CBA-H scales, or stronger intensities of symptoms, as revealed by contingency tables for the DHI test and by the comparisons of medians and interquartile intervals for the SVQ questionnaire (see Supplementary Materials).

**Figure 3:**
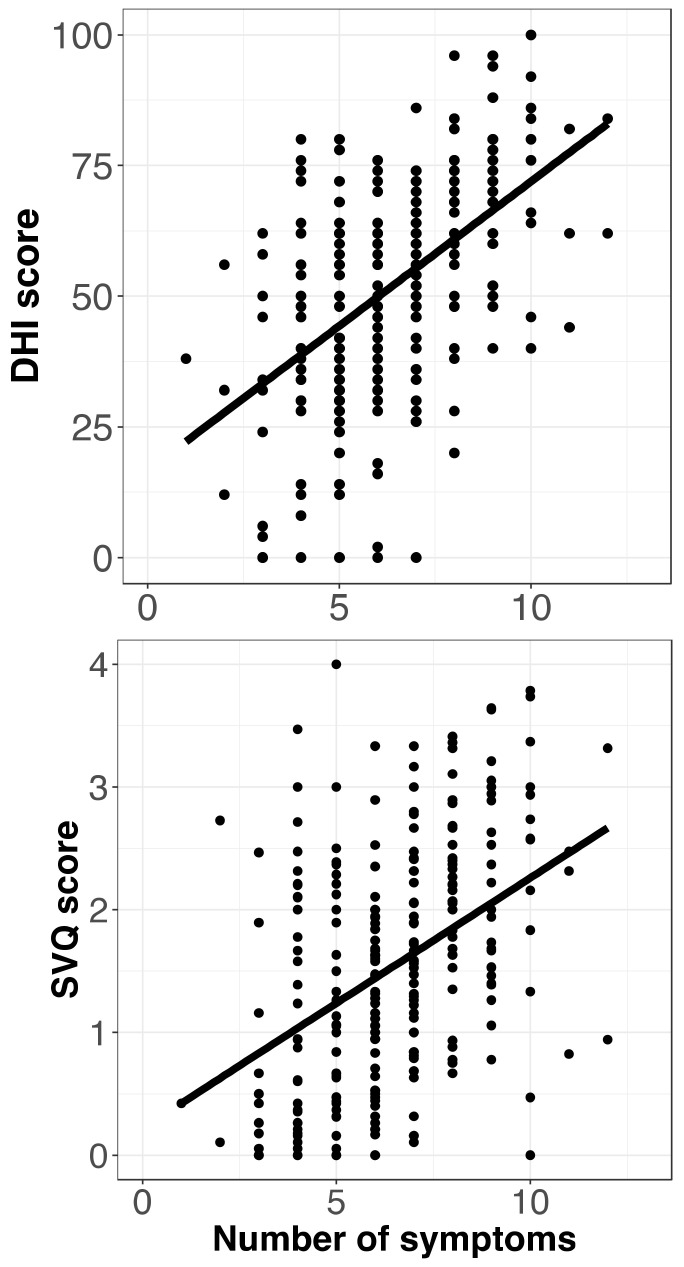
Scatter plots of the Dizziness Handicap Inventory (DHI) overall scores (**top**) and the Situational Vertigo Questionnaire (SVQ) scores (**bottom**) against the number of symptoms reported by FM participants. Regression lines are also shown.

A significant correlation was also observed between DHI scores and the number of symptoms reported by FM patients, while a similar result was found with the SVQ test (R^2^ = 0.281 and 0.177 for DHI and SVQ, respectively, *p* < 0.001) (Figure 3).

Regarding early-life stressors, 43% of FM participants reported childhood/adolescence trauma, including physical, emotional, and sexual abuse, emotional and physical neglect, loss of a parent, and economic hardships. Accordingly, negative correlations concerning the psychological sphere were also found between DHI overall scores and the evaluation rates attributed by FM participants to their childhood or adolescence, obtaining ρ_s_ = −0.20 and ρ_s_ = −0.27 (*p* < 0.001), respectively. The same analysis for the SVQ test yielded ρ_s_ = −0.23 and ρ_s_ = −0.25 (*p* < 0.001), for the evaluation rates of childhood and adolescence, respectively.

### 3.6. Multivariate Analysis of DHI and SVQ Data

Based on DHI and SVQ data, a Cluster Analysis was conducted on FM participants and an Exploratory Factor Analysis on the DHI and SVQ items.

#### 3.6.1. Analysis of Participants

**Figure 4:**
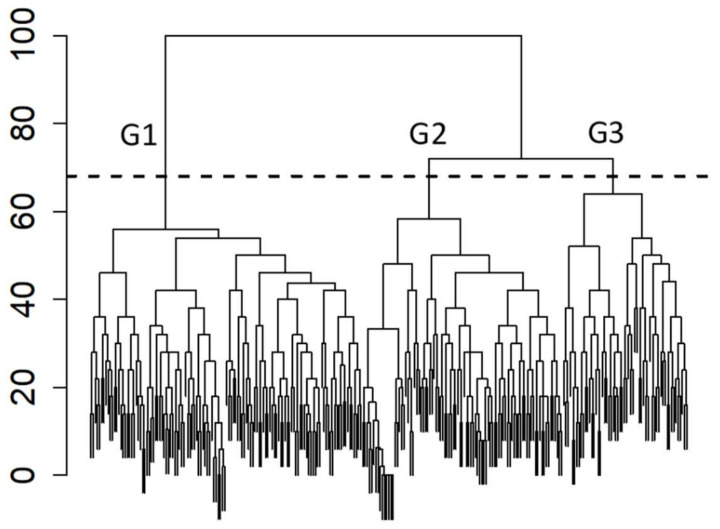
Cluster dendrogram of Fibromyalgia patients obtained from a multivariate analysis of Dizziness Handicap Inventory data (complete linkage agglomerative clustering with Manhattan distance). The height of each node (vertical axis) is the distance value between the right and left sub-branch clusters. The dendrogram reveals the presence of three groups designated Group 1 (G1), Group 2 (G2) and Group 3 (G3).

**Figure 5:**
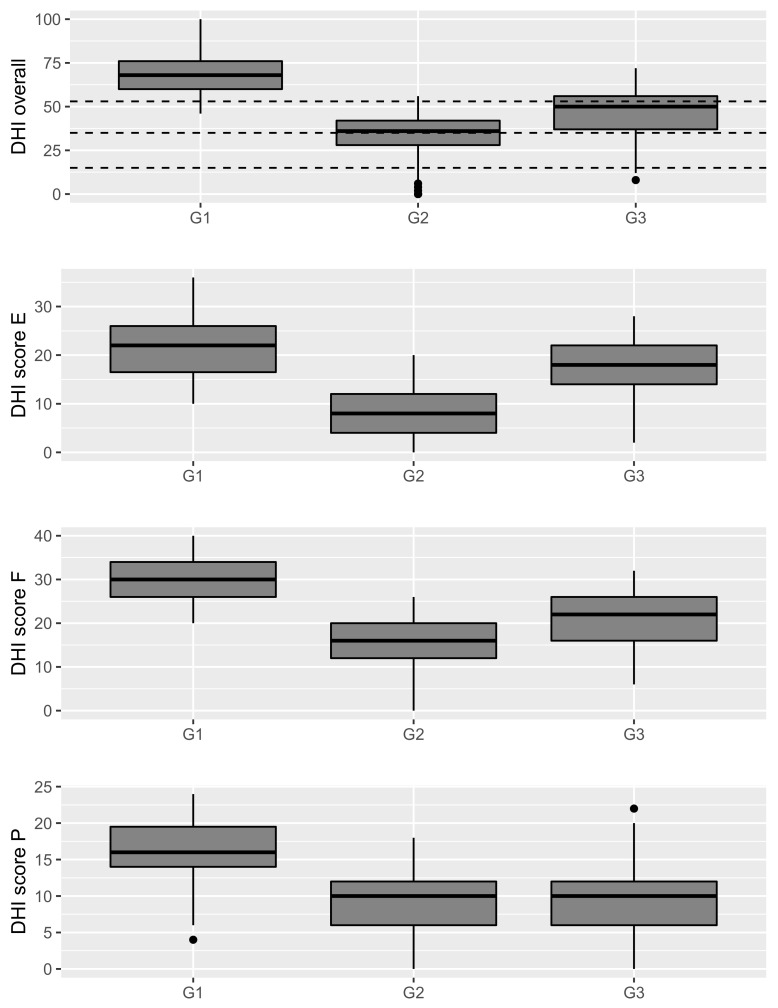
Boxplot charts of Dizziness Handicap Inventory (DHI) overall scores and DHI component scores (E = Emotional, F = Functional, P = Physical), classified by the three groups (Group 1 (G1), Group 2 (G2) and Group 3 (G3)) obtained from the Cluster Analysis (see Figure 4). In the top boxplot, horizontal lines indicate the DHI cutoffs for the four handicap levels.

In the Cluster Analysis of DHI data, the participants were clustered into three groups, indicated by G1, G2, and G3, containing respectively 126, 93, and 58 subjects (Figure 4). The DHI overall score distributions classified by the three groups of Cluster Analysis were significantly different (Kruskal–Wallis test, *p* < 0.01), revealing highest values in G1, lowest values in G2, and intermediate values in G3 (Figure 5). The score distributions of each DHI component (Emotional, Functional, and Physical) also showed highest values in the G1 group, lowest in G2, and intermediate in G3 for the Emotional and Functional components, whereas in the Physical component, the score distributions of G2 and G3 were lower than G1 but did not differ from each other (Figure 5). Such a complex of data indicates that the sharpest separation is between the subjects with severe handicap, i.e., G1 with DHI score equal to 54 or higher, and the rest of subjects. The G2 and G3 groups showed a lesser degree of separation, but if cross correlations with symptoms were considered, a sharp difference was found especially for dizziness (average scores, G1 = 0.74; G2 = 0.49; G3 = 0.64), migraine (G1 = 0.75; G2 = 0.64; G3 = 0.50), and depression (G1 = 0.52; G2 = 0.20; G3 = 0.43), while among other parameters, the sharpest difference was found with diagnosis delay (median value in years, G1 = 4; G2 = 2; G3 = 4).

Considering the SVQ data, in the Cluster Analysis, a single cluster of patients was found (not shown).

#### 3.6.2. Analysis of Questionnaire Items

**Figure 6:**
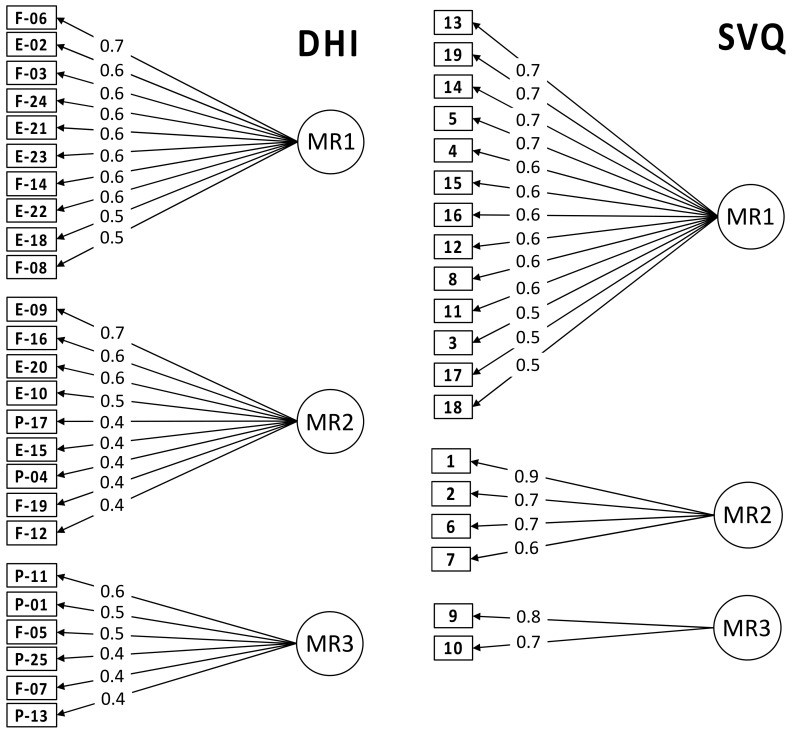
Factor structures derived from the Exploratory Factor Analysis of the Dizziness Handicap Inventory (DHI) (**left**) and Situational Vertigo Questionnaire (SVQ) (**right**) items. A significant three-factor structure (MR1, MR2, and MR3, MR = minimum residual) was derived for the DHI items. The arrows represent the best item-factor association, and the numbers represent the loadings. In the analysis of DHI data, the letters E, F, and P indicate the Emotional, Functional, and Physical components of the test, respectively.

The Exploratory Factor Analysis of the DHI items revealed three main factors. The first one includes Emotional and Functional items only, the second one mainly Emotional and Functional items but also two Physical items, while the third one includes mainly Physical items and two Functional items (Figure 6). These factors do not correspond to the three conventional components of the test, namely the Functional, Emotional, and Physical one, as confirmed by Confirmatory Factor Analysis that rejected the factorization based on the DHI conventional components with a value of the Tucker–Lewis Index (TLI) = 0.736 (TLI ranges between 0 and 1, good fit is indicated by TLI > 0.95). A similar analysis of the different dimensions of disability and handicap of the DHI test, conducted on dizziness patients, also yielded three factors with a mixed composition of the E, F, and P components [33]. However, also the factors created by this latter analysis did not correspond to those derived from the questionnaire data of our FM population (TLI = 0.748). Hence, the vestibular pattern emerging from our FM patients can be distinguished from the structure of the DHI test and from the pattern of subjects affected by dizziness but not classified as FM patients.

As for SVQ data, the Exploratory Factor Analysis showed that most questionnaire items were combined into the first factor accounting for the highest amount of variance (Figure 6). Hence, this multifactorial analysis revealed that most of the SVQ variability is explained by a single significant factor.

## 4. Discussion

### 4.1. Presence of Vestibular Symptoms in FM

The FM patients that participated in this study were representative of the FM population, considering the cluster of symptoms (especially fatigue and sleep disturbances) [34], female prevalence [35], and general clinical features [36,37], as well as the long diagnostic delay and duration of the disease [38]. Moreover, the age of female participants at the time of questionnaire completion and at the onset of the condition were in agreement with the known perimenopausal and menopausal phase as a potential cause or aggravation of symptoms [37] and with the hypothesis that there is an association between phasic fluctuations of progesterone and functional symptoms in FM [39].

The first and primary results obtained from our data was the demonstration of a higher prevalence of perceived handicap resulting from dizziness in FM participants, with respect to control subjects. In addition, a parallel association was observed between DHI and SVQ scores with pain symptoms as well as with some non-pain symptoms. This clearly suggests that both vestibular problems and visual vestibular mismatch are potentially inherent to the FM syndrome. Such a result supports the view that FM should not be considered tout court a chronic pain problem, but rather a sensory processing disturbance involving different sensorial pathways [3].

### 4.2. Peculiarity of FM Dizziness Symptoms

A significant result of the cluster analysis of patients based on DHI items was the finding of three groups, with a main segregation of subjects with severe handicaps. This suggests that vestibular problems in FM participants do not represent a continuum but can be subdivided into distinct patterns. As a reinforcement of this view, the factor analysis yielded a peculiar structuring of the DHI handicap factors, not corresponding to the canonical components, i.e., Emotional, Functional, and Physical ones. These findings suggest that FM dizziness may have peculiar features that distinguish it from other dizziness disorders, raising the need for more investigations to clarify if this dizziness is part of the FM symptom cascade, i.e., a psychosomatic component, or a direct consequence of central sensory processing disorder [40].

When considering multifactorial analysis of SVQ data, no specific structure in either patients or items was observed. In 2009, the Bárány International Society for Neuro-Otology introduced Visual Induced Dizziness (VID) to describe symptoms like those of the SVQ questionnaire and recently included them under the umbrella of Persistent Postural-Perceptual Dizziness (PPPD) [21]. Our data suggest that VID may be present in FM patients, but also in this case, further analysis is required to understand if it is the result of a central dizziness disorder, like PPPD, or a psychosomatic issue. In this respect, clues about the mechanism of dizziness in FM can be found in the widespread occurrence of fatigue and sleep disorders, suggesting that FM patients are subjected to migraine and potentially to dysautonomia. A correlation between dysautonomia and vestibular problems has been recognized for PPPD, which similarly to FM is characterized by dizziness, fatigue, and secondary mood disorders [41,42,43]. Moreover, dysautonomia has been considered to fall into the above mentioned “central sensitivity syndromes” [8]. Hence, FM could be associated with specific types of dizziness occurring in dysautonomic conditions, like for example the one experienced by patients suffering from Postural Orthostatic Tachycardia Syndrome (POTS) [44].

### 4.3. Involvement of Cognitive and Behavioral Impairment

The observed correlations between DHI and psychophysical stress/wellbeing, depressive mood, state anxiety, and health-care related fears could be a reasonable expectation in pathological subjects. However, the inability of FM patients to relax is in line with their high rate of neurovegetative symptoms, such as fatigue, sleep disturbances, dizziness, and migraine, deserving further investigation and a more accurate clinical consideration. In line with previous studies, our data highlights the need for a psychological evaluation of FM patients with dizziness handicap, since emotional instability could negatively affect therapeutic outcomes [45,46]. Moreover, the correlation between dizziness handicap and neuroticism is particularly relevant since neuroticism confers stress vulnerability [47] while chronic stress is associated with both depressive mood and FM [48,49,50]. This is further highlighted by the negative correlation between the rating of childhood or adolescence by FM participants to our study and their DHI score, since chronic stress, particularly early life stress, is a candidate common mechanism underlying FM and vestibular disorders. In line with this, 43% of FM respondents reported early-life adversities [51]. Taking this into consideration, our results suggest the need to further explore the etiology of dizziness as well as consider psychological adversity in the development of central vestibular symptoms.

When considering the SVQ and CBA-H, the correlation with depressive mood and reactions was confirmed, even though it was lower than in DHI, possibly because SVQ items refer to visual and spatial skills rather than to functional and emotional aspects. Despite early-life stressors encompassing a wide range of events such as physical, emotional and sexual abuse, emotional and physical neglect, family bereavements, and poverty, they all affect the developing brain through possible common mechanisms that are able to unbalance the excitatory/inhibitory neurotransmission [23]. As a result, for future research, such stressors should be considered when examining FM patients as well as patients affected by central vestibular disorders (e.g., Mal de Debarquement Syndrome, Vestibular Migraine, PPPD). It is known that elevated levels of stress and anxiety often accompany vestibular dysfunction and that dizziness and loss of balance is observed in patients with anxiety. Hence, when considering the FM population, stress and psychological components should be equally considered as well as their potential vestibular compensation [26]. The effect of stress on the neurochemical imbalances leading to dizziness should be further explored [43,52]. This would allow a full understanding of the nature of dizziness affecting FM patients and ultimately improve their management.

In summary, our questionnaire data showed a prevalence of mild, moderate, and mostly severe handicap caused by dizziness and vestibular symptoms (VID) in FM participants versus a control group. This kind of symptomatology was characterized from a broad perspective, including associations with pain, non-pain, and psychological symptoms. Our data are in line with the idea that FM falls into the cluster of central sensory disorders and provide a strong indication that FM patients should be screened for dizziness. This further highlights the main issue observed in the referral pathway of these patients, where neurologists and otologists still play a secondary role [53].

One limitation to our study was the usage of generic tools such as DHI and SVQ questionnaires for detecting dizziness. Future studies should implement more specific tools for FM patients such as the 20-item Joint Assessment of Equilibrium and in addition the Neuro-Motor Function Screening, which has been recently validated as a reliable tool for assessing balance in patients with FM [54].

## 5. Conclusions

Our study showed that:

- FM participants scored high in self-perceived handicap associated with dizziness and VID, despite a low number of vestibular diagnoses;
- vestibular symptoms were correlated with different FM pain and non-pain symptom scales, suggesting we should further explore the correlation between stress and psychological symptoms in relation to dizziness;
- the pattern of symptoms other than pain suggested a prevalence of migraine and dysautonomia, possibly co-existing with central vestibular dysfunctions;
- data revealed an impact of dizziness on the subject emotional status, possibly involving a noxious self-sustained chronic stress condition, and raising the need for including stress and psychological components when considering vestibular symptoms in FM patients.

In conclusion, we have shown the presence of an overt dizziness condition in FM patients, deserving careful clinical attention due to its possible inherent role in the disease.

## Figures and Tables

**Table 1:** Percent distribution of demographics in Fibromyalgia (FM) participants (*n* = 277) and of gender in Controls (*n* = 80).

**Gender**	Female	Male	No answer			
FM	89.5 *	4.0 *	6.5			
Control	81.3 ^#^	18.7 ^#^	0			
**Education**	Primary	Lower secondary	Upper secondary	Academic degree	PhD or equivalent	
	1.1	17.3	55.6	18.4	7.6	
**Marital status**	Single	Married/cohabitant	Separated/divorced	Widowed		
	20.9	60.7	16.6	1.8		
**Employment**	Grey-collar	White-collar	Blue-collar	Shopkeeper	Unemployed	No answer
	11.5	35.0	13.7	4.7	34	1.1

*, # = chi-squared test, *p* < 0.01.

**Table 2:** Descriptive statistics of clinical features in Fibromyalgia (FM) participants (*n* = 277) and of age in Controls (*n* = 80).

		Q1	Median	Mean ± SD	Q3
Patient age (years)	FM	41	50 *	48.1 ± 10.7	56
	controls	35	42 *	46.9 ± 13.5	60
Height (cm)		160	164	164 ± 6.3	168
Weight (Kg)		58	66	68.2 ± 15.2	77
Body Mass Index (BMI)		21.2	24.2	25.3 ± 5.3	28.4
Age-of-onset (years)		30	39	37.2 ± 11	45
Disease duration (years)		5	9	11.9 ± 9.3	16
Diagnostic delay (years)		1	4	6.35 ± 7.9	8

Q1 = first quartile; Q3 = third quartile; * = Wilcoxon test, *p* > 0.05.

**Table 3:** Frequency of non-pain symptoms reported in the questionnaire by the Fibromyalgia patients (*n* = 277).

Symptom	Percent Frequency
Fatigue	98.6
Sleep disturbance	89.9
Brain fog	81
Migraine	65.8
Dizziness	63.6
Anxiety	63.3
Photophobia	51.3
Depression	39.6
Nausea	37.8
Diplopia	33.4
Gut disorders	12
Tinnitus	5.8

**Table 4:** Descriptive statistics of the Dizziness Handicap Inventory (DHI) and the Situational Vertigo Questionnaire (SVQ) scores obtained by Fibromyalgia (FM) patients and control subjects.

Score	Group	Min	Q1	Median	Mean	Q3	Max
DHI	FM	0	38	54 *	52	66	100
	Control	0	0	2 *	7.2	8	82
							
SVQ	FM	0	0.78	1.53 ^#^	1.53	2.22	4
	Control	0	0	0.19 ^#^	0.41	0.44	2.81

Q1 = first quartile; Q3 = third quartile; *, # = *p* < 0.001 Wilcoxon rank sum test.

**Table 5:** Values of Spearman’s rank correlation ρ_s_ between either Dizziness Handicap Inventory (DHI) or the Situational Vertigo Questionnaire (SVQ) scores and the scores of pain types as defined in the questionnaire.

Pain Types	DHI	SVQ
Occasional pain	0.395 (*p* < 0.001)	0.302 (*p* < 0.001)
Pressure pain	0.328 (*p* < 0.001)	0.217 (*p* < 0.001)
Numbness	0.351 (*p* < 0.001)	0.285 (*p* < 0.001)
Burning	0.348 (*p* < 0.001)	0.244 (*p* < 0.001)
Tingling	0.347 (*p* < 0.001)	0.319 (*p* < 0.001)
Light contact	0.347 (*p* < 0.001)	0.299 (*p* < 0.001)
Sudden pain	0.264 (*p* < 0.001)	0.236 (*p* < 0.001)

*p*-values for significance tests are shown in parentheses.

## Data Availability

The data presented in this study are available within the article and in Supplementary Materials.

## Supplementary Materials

The following supporting information can be downloaded at: https://www.mdpi.com/article/10.3390/jcm11144017/s1.
