# Supplementary material for: Vestibular Disability/Handicap in Fibromyalgia: A Questionnaire Study
Source: J Clin Med. 2022 Jul 11;11(14):4017. doi: 10.3390/jcm11144017 (PMC9315683; doi:10.3390/jcm11144017)
Supplement: Supplementary file 1 [file jcm-11-04017-s001.zip › jcm-1754821-supplementary.pdf]

Vestibular Disability/Handicap in Fibromyalgia: A Questionnaire Study

Viviana Mucci [1], Ilaria Demori [2,*], Fabio Rapallo [3], Elena Molinari [4], Serena Losacco [5], Lucio Marinelli [6,7], Cherylea J. Browne [1] and Bruno Burlando [5]

1 School of Science, Western Sydney University, Sydney, NSW 2751, Australia; viviana.mucci@gmail.com or v.mucci@westernsydney.edu.au (V.M.); c.browne@westernsydney.edu.au (C.J.B.)
2 Department of Earth, Environmental and Life Sciences (DISTAV), University of Genova, Corso Europa, 26, 16132 Genova, Italy
3 Department of Economics (DIEC), University of Genova, Via Vivaldi, 5, 16126 Genova, Italy; fabio.rapallo@unige.it
4 Clinical Psychology Unit, E.O. Ospedali Galliera, Via Mura delle Cappuccine 14, 16128 Genova, Italy; elena.molinari@galliera.it
5 Department of Pharmacy, DIFAR, University of Genova, Viale Benedetto XV, 3, 16132 Genova, Italy; losacco@difar.unige.it (S.L.); bruno.pietro.burlando@unige.it (B.B.)
6 Department of Neuroscience, Rehabilitation, Ophthalmology, Genetics, Maternal and Child Health,
DINOGMI, University of Genova, Largo P. Daneo 3, 16132 Genova, Italy; lucio.marinelli@unige.it
7 IRCCS Ospedale Policlinico San Martino, Department of Neuroscience, Division of Clinical Neurophysiology, Largo R. Benzi 10, 16132 Genova, Italy
* Correspondence: idemori@unige.it

Supplementary data

Analysis of DHI scores

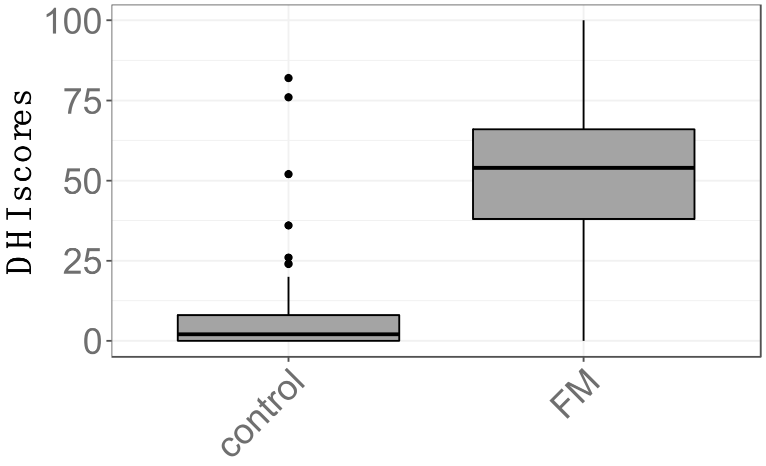

Comparison of total DHI scores between FM and controls

	Min.	1st Qu.	Median	Mean	3rd Qu.	Max.
FM	0.00	38.00	54.00	51.99	66.00	100.00
controls	0.00	0.00	2.00	7.16	8.00	82.00

N=277 valid responses in the FM sample
N=80 valid responses in the controls sample

Wilcoxon rank sum test with continuity correction
W = 21203, p-value < 2.2e-16

Percent distribution of subjects among the different handicap levels of the DHI test

	no handicap	mild handicap	moderate handicap	severe handicap
FM	5.8	15.5	28.5	50.2
controls	89.5	5.5	2.5	2.5

Comparison of the scores of the different DHI components between FM and controls

DHI component		1st Qu.	Median	3rd Qu.	p value*
E	FM	38	54	66	< 0.0001
	controls	0	2	8	
F	FM	16	24	30	< 0.0001
	controls	0	0	2	
P	FM	8	12	16	< 0.0001
	controls	0	0	4	

E = Emotional; F = Functional; P = Physical; * = Wilcoxon rank sum test

Correlations between DHI scores and painDETECT questionnaire scores (Spearman's rank correlation coefficient).
DHI overall scores: 0.442577
DHI E component: 0.3339791
DHI F component: 0.4503229
DHI P component: 0.3980511

Correlations between the DHI overall scores and pain intensity scores (Spearman's rank correlation coefficient).
rho.s = 0.3464907

Correlations between DHI scores and the scores of each pain type (Spearman's rank correlation coefficient)
DHI overall scores
Pressure pain: 0.3284688
Occasional pain: 0.3950016
Burning: 0.3480615
Numbness: 0.3505187
Sudden pain: 0.2640404
Tingling: 0.3472928
Light contact: 0.3472884

DHI E scores
Pressure pain: 0.2535965
Occasional pain: 0.286041
Burning: 0.2612536
Numbness: 0.2689884
Sudden pain: 0.1980363
Tingling: 0.2618997
Light contact: 0.2475746

DHI F scores
Pressure pain: 0.325775
Occasional pain: 0.3864555
Burning: 0.3342964
Numbness: 0.350324
Sudden pain: 0.256503
Tingling: 0.3481341
Light contact: 0.3268056

DHI P scores
Pressure pain: 0.2751862
Occasional pain: 0.3751805
Burning: 0.3478595
Numbness: 0.3196578

Sudden pain: 0.2655094
Tingling: 0.3170133
Light contact: 0.3544598

Mean FM diagnosis delays in years within the Handicap levels of the DHI overall scores

0 No Handicap	1 Mild Handicap	2 Moderate Handicap	3 Severe Handicap
3.466667	5.097561	5.985915	6.723577

Correlations between DHI overall scores and the scores assigned by patients to their childhood and youth periods (Spearman's rank correlation coefficient).
Childhood: -0.2011472
Youth: -0.267147

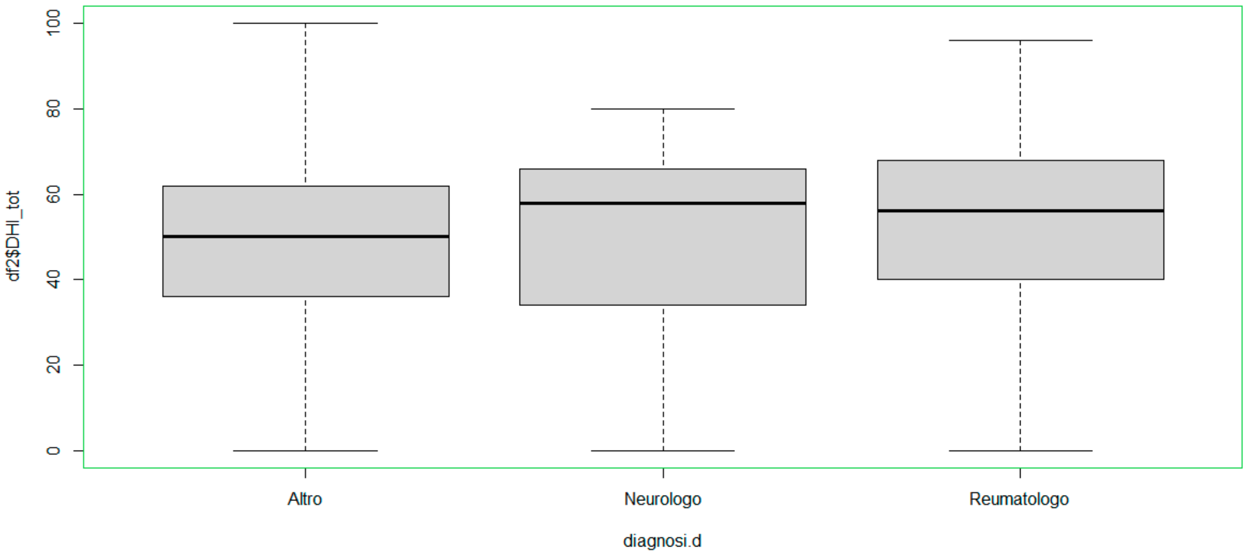

Boxplot charts of DHI overall scores in patients subdivided according to the source of FM diagnosis. Left: other; middle: neurologist; right: rheumatologist.

Number of patients according to their diagnosis

Other	Neurologist	Rheumatologist
81	9	187

Contingency tables between the Handicap levels of DHI overall scores and the items of the CBA-H test expressed as absence/presence (FALSE/TRUE) of the corresponding psychological condition (Pearson's Chi-squared test)
Anxiety

	FALSE	TRUE
0 No Handicap	12	4
1 Mild Handicap	28	15

	FALSE	TRUE
2 Moderate Handicap	48	31
3 Severe Handicap	53	86

X-squared = 19.339, df = 3, p-value = 0.0002326

Health-care related fears

	FALSE	TRUE
0 No Handicap	15	1
1 Mild Handicap	41	2
2 Moderate Handicap	69	10
3 Severe Handicap	107	32

X-squared = 10.861, df = 3, p-value = 0.0125

Depression

	FALSE	TRUE
0 No Handicap	15	1
1 Mild Handicap	40	3
2 Moderate Handicap	60	19
3 Severe Handicap	73	66

X-squared = 34.778, df = 3, p-value = 1.357e-07

Emotional instability-Depressive mood

	FALSE	TRUE
0 No Handicap	11	5
1 Mild Handicap	14	29
2 Moderate Handicap	19	60
3 Severe Handicap	10	129

X-squared = 43.854, df = 3, p-value = 1.621e-09

Psychophysical wellbeing (TRUE = absence of condition)

	FALSE	TRUE
0 No Handicap	3	13
1 Mild Handicap	2	41
2 Moderate Handicap	5	74
3 Severe Handicap	2	137

X-squared = 11.603, df = 3, p-value = 0.008873

Perceived psychophysical stress

	FALSE	TRUE
0 No Handicap	14	2
1 Mild Handicap	38	5
2 Moderate Handicap	57	22
3 Severe Handicap	89	50

X-squared = 11.836, df = 3, p-value = 0.007966

Neuroticism

	FALSE	TRUE
0 No Handicap	8	8
1 Mild Handicap	37	6

	FALSE	TRUE
2 Moderate Handicap	49	30
3 Severe Handicap	82	57

X-squared = 11.987, df = 3, p-value = 0.007429

Introversion/extroversion (TRUE = tendency to introversion)

	FALSE	TRUE
0 No Handicap	3	13
1 Mild Handicap	12	31
2 Moderate Handicap	30	49
3 Severe Handicap	59	80

X-squared = 5.5439, df = 3, p-value = 0.136

Social anxiety

	FALSE	TRUE
0 No Handicap	12	4
1 Mild Handicap	33	10
2 Moderate Handicap	59	20
3 Severe Handicap	80	59

X-squared = 9.9015, df = 3, p-value = 0.01942

Haste and Impatience

	FALSE	TRUE
0 No Handicap	10	6
1 Mild Handicap	31	12
2 Moderate Handicap	42	37
3 Severe Handicap	63	76

X-squared = 10.145, df = 3, p-value = 0.01737

Excessive Involvement

	FALSE	TRUE
0 No Handicap	9	7
1 Mild Handicap	23	20
2 Moderate Handicap	40	39
3 Severe Handicap	59	80

X-squared = 2.8407, df = 3, p-value = 0.4168

Hostility

	FALSE	TRUE
0 No Handicap	16	0
1 Mild Handicap	43	0
2 Moderate Handicap	79	0
3 Severe Handicap	132	7

X-squared = 7.1298, df = 3, p-value = 0.06787

Inability to Relax

	FALSE	TRUE
0 No Handicap	2	14
1 Mild Handicap	7	36

2 Moderate Handicap	26	53
3 Severe Handicap	72	67

X-squared = 24.927, df = 3, p-value = 1.599e-05

Interpersonal Difficulties

	FALSE	TRUE
0 No Handicap	13	3
1 Mild Handicap	36	7
2 Moderate Handicap	58	21
3 Severe Handicap	67	72

X-squared = 26.885, df = 3, p-value = 6.224e-06

Leadership/Competitiveness

	FALSE	TRUE
0 No Handicap	9	7
1 Mild Handicap	26	17
2 Moderate Handicap	54	25
3 Severe Handicap	84	55

X-squared = 1.7505, df = 3, p-value = 0.6258

Irritability

	FALSE	TRUE
0 No Handicap	6	10
1 Mild Handicap	11	32
2 Moderate Handicap	26	53
3 Severe Handicap	38	101

X-squared = 1.5634, df = 3, p-value = 0.6677

Contingency tables between the Handicap levels of DHI overall scores and the absence/presence (0/1) of symptoms other than pain (Pearson's Chi-squared test)

Brain fog	0	1
0 No Handicap	6	10
1 Mild Handicap	10	32
2 Moderate Handicap	15	63
3 Severe Handicap	21	118

X-squared = 5.5793, df = 3, p-value = 0.134

Dizziness	0	1
0 No Handicap	15	1
1 Mild Handicap	20	22
2 Moderate Handicap	30	48
3 Severe Handicap	35	104

X-squared = 32.731, df = 3, p-value = 3.67e-07

Migraine	0	1
0 No Handicap	11	5
1 Mild Handicap	16	26

2 Moderate Handicap 29 49
3 Severe Handicap 38 101
X-squared = 11.989, df = 3, p-value = 0.007419

Anxiety 0 1
0 No Handicap 8 8
1 Mild Handicap 19 23
2 Moderate Handicap 29 49
3 Severe Handicap 45 94
X-squared = 3.6624, df = 3, p-value = 0.3003

Photophobia 0 1
0 No Handicap 10 6
1 Mild Handicap 26 16
2 Moderate Handicap 40 38
3 Severe Handicap 58 81
X-squared = 7.0644, df = 3, p-value = 0.06987

Depression 0 1
0 No Handicap 13 3
1 Mild Handicap 35 7
2 Moderate Handicap 54 24
3 Severe Handicap 64 75
X-squared = 26.656, df = 3, p-value = 6.95e-06

Nausea 0 1
0 No Handicap 12 4
1 Mild Handicap 31 11
2 Moderate Handicap 50 28
3 Severe Handicap 78 61
X-squared = 5.8305, df = 3, p-value = 0.1202

Diplopia 0 1
0 No Handicap 14 2
1 Mild Handicap 37 5
2 Moderate Handicap 54 24
3 Severe Handicap 78 61
X-squared = 18.962, df = 3, p-value = 0.0002784

Gut disorders 0 1
0 No Handicap 15 1
1 Mild Handicap 40 2
2 Moderate Handicap 72 6
3 Severe Handicap 115 24
X-squared = 7.6057, df = 3, p-value = 0.0549

Tinnitus 0 1
0 No Handicap 16 0

1 Mild Handicap 37 5
2 Moderate Handicap 72 6
3 Severe Handicap 134 5
X-squared = 5.5792, df = 3, p-value = 0.134

Fatigue	0	1
0 No Handicap	1	15
1 Mild Handicap	2	41
2 Moderate Handicap	0	79
3 Severe Handicap	1	138

X-squared = 7.3747, df = 3, p-value = 0.06087

Sleep disturbance	0	1
0 No Handicap	3	13
1 Mild Handicap	6	37
2 Moderate Handicap	11	68
3 Severe Handicap	8	131

X-squared = 6.1791, df = 3, p-value = 0.1032

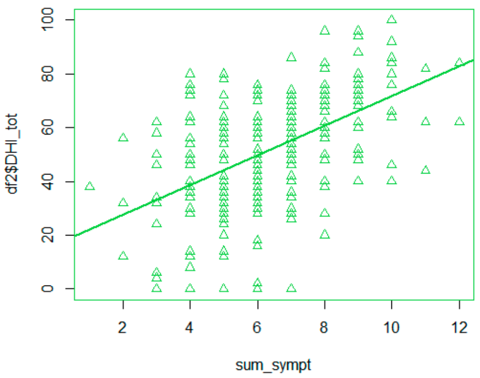

Plot of DHI overall scores vs. the number of reported symptoms other than pain

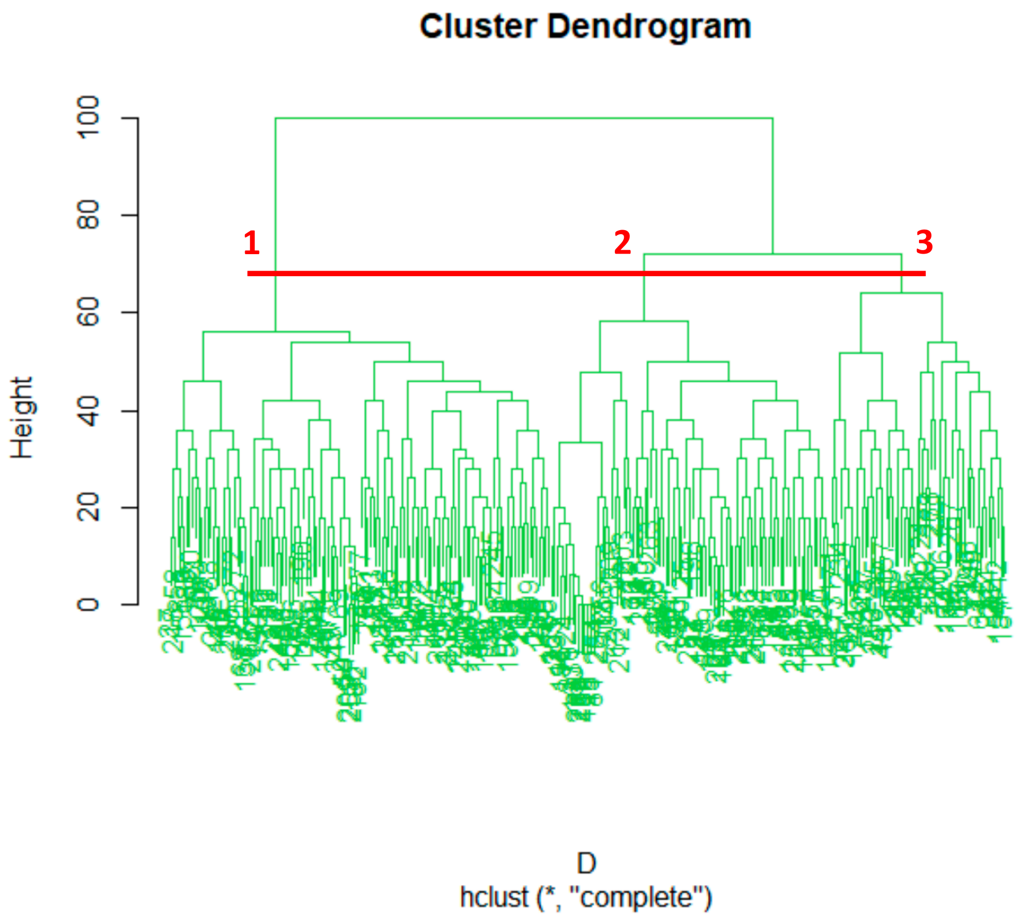

Cluster analysis of FM patients with Manhattan distance and complete linkage aggregation method

Number of subjects in each group

1	2	3
126	93	58

Number of subjects in each group subdivided into the Handicap levels of the DHI test

	grp		
	1	2	3
0 No Handicap	0	14	2
1 Mild Handicap	0	31	12
2 Moderate Handicap	11	46	22
3 Severe Handicap	115	2	22

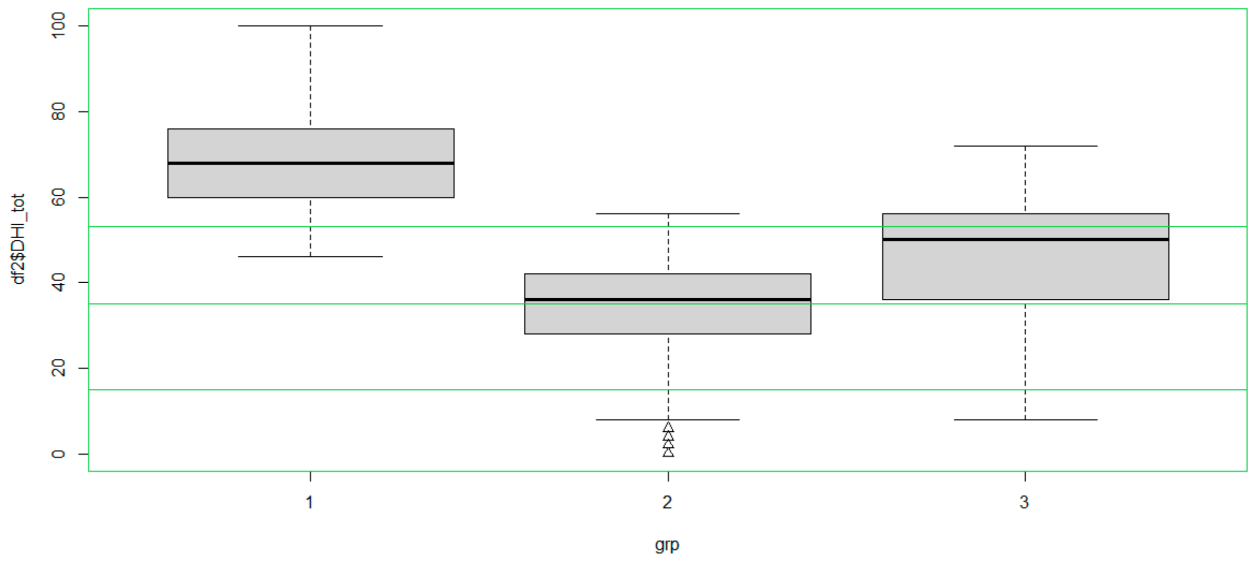

Boxplot charts of the DHI overall scores in the three groups of the cluster analysis

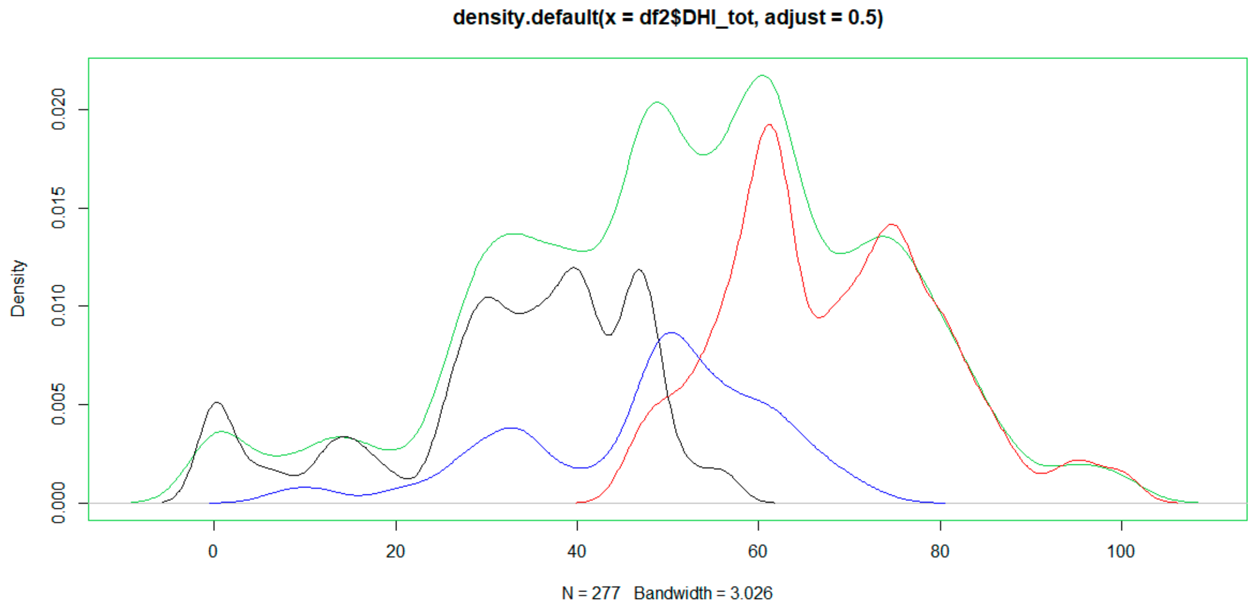

Density estimation with Gaussian kernel and optimal bandwith concerning DHI overall scores. Green line: total sample; black line: group 1; red line: group 2; blue line: group 3.

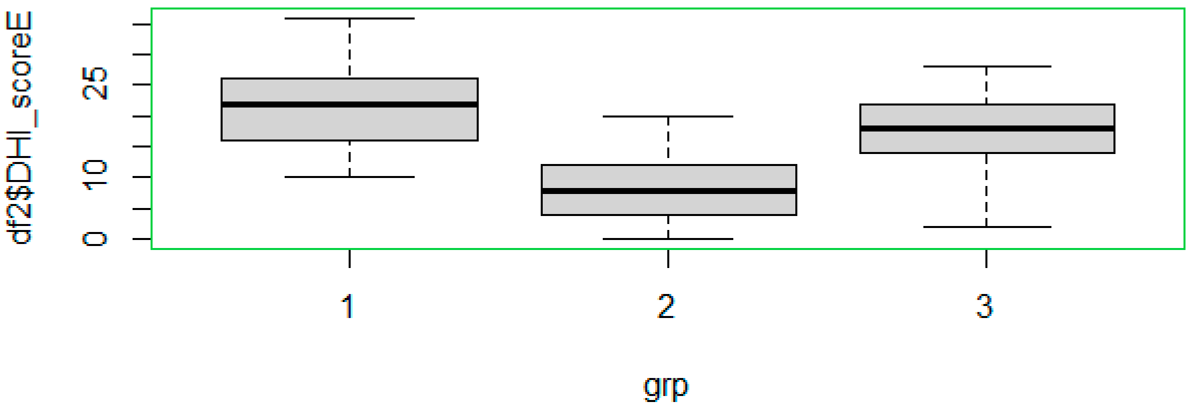

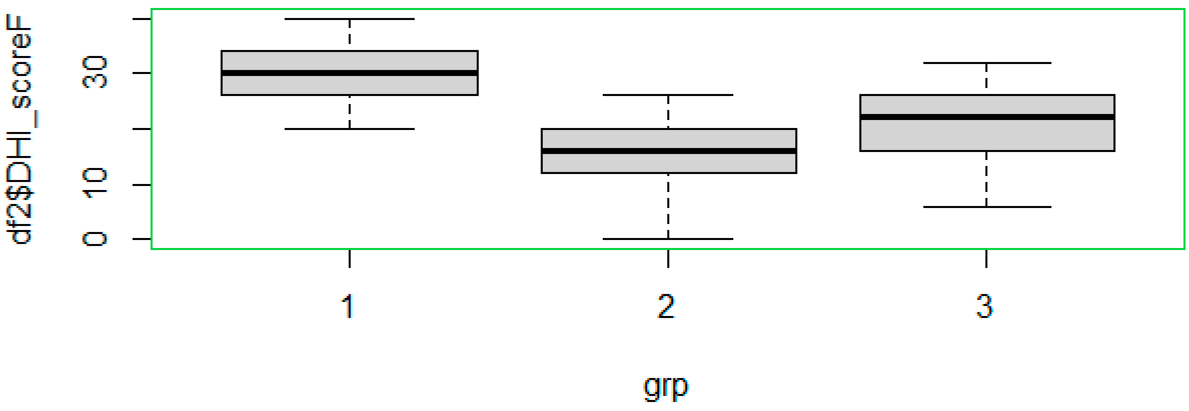

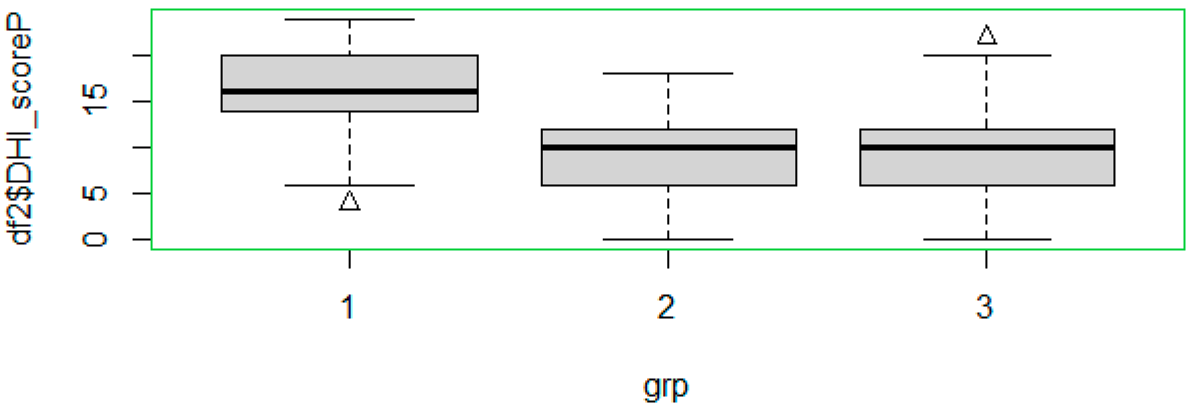

Boxplot charts of the three DHI components E, F, and P scores in the three groups of the cluster analysis,

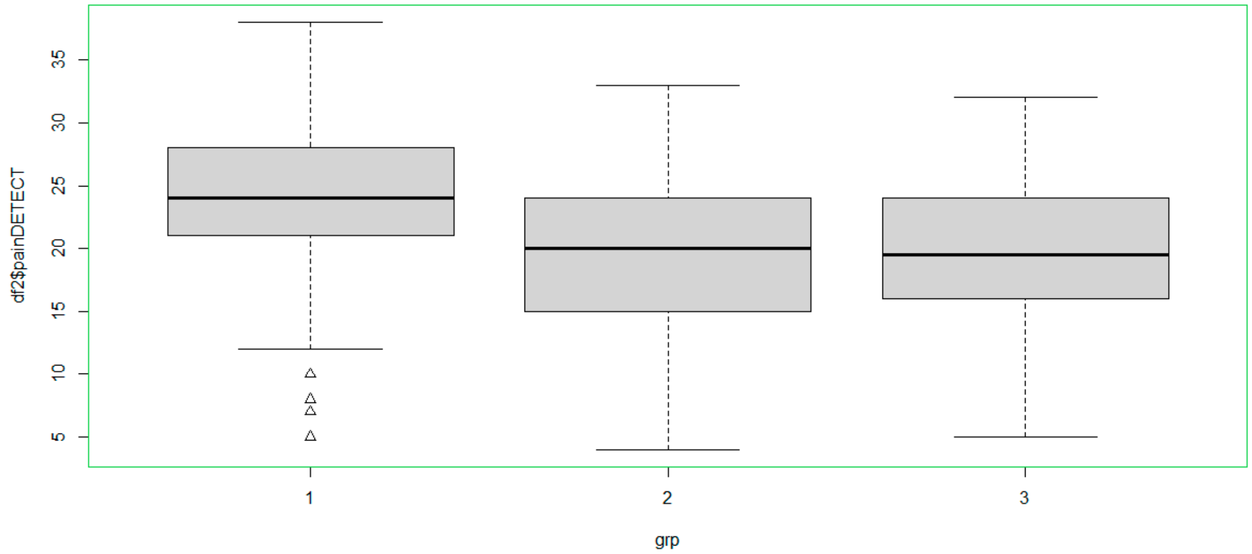

Boxplot charts of the painDETECT questionnaire scores in the three groups of the cluster analysis.

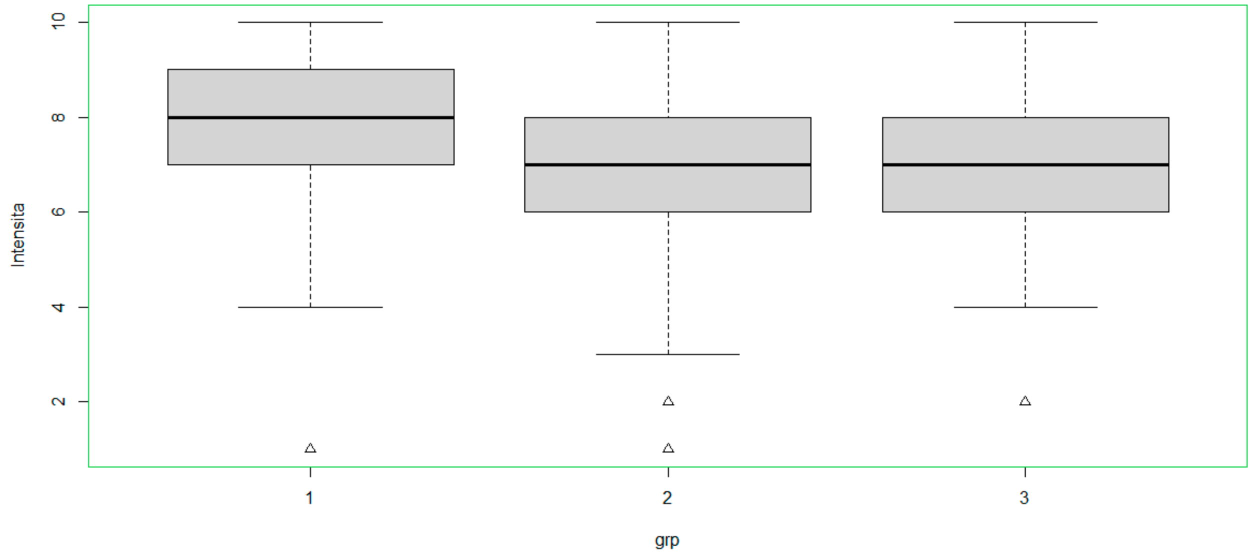

Boxplot charts of the Pain Intensity scores in the three groups of the cluster analysis.

Mean delays in years of FM diagnosis within the three groups of the cluster analysis

1	2	3
6.414414	4.678161	7.576923

Exploratory Factor Analysis of the DHI items

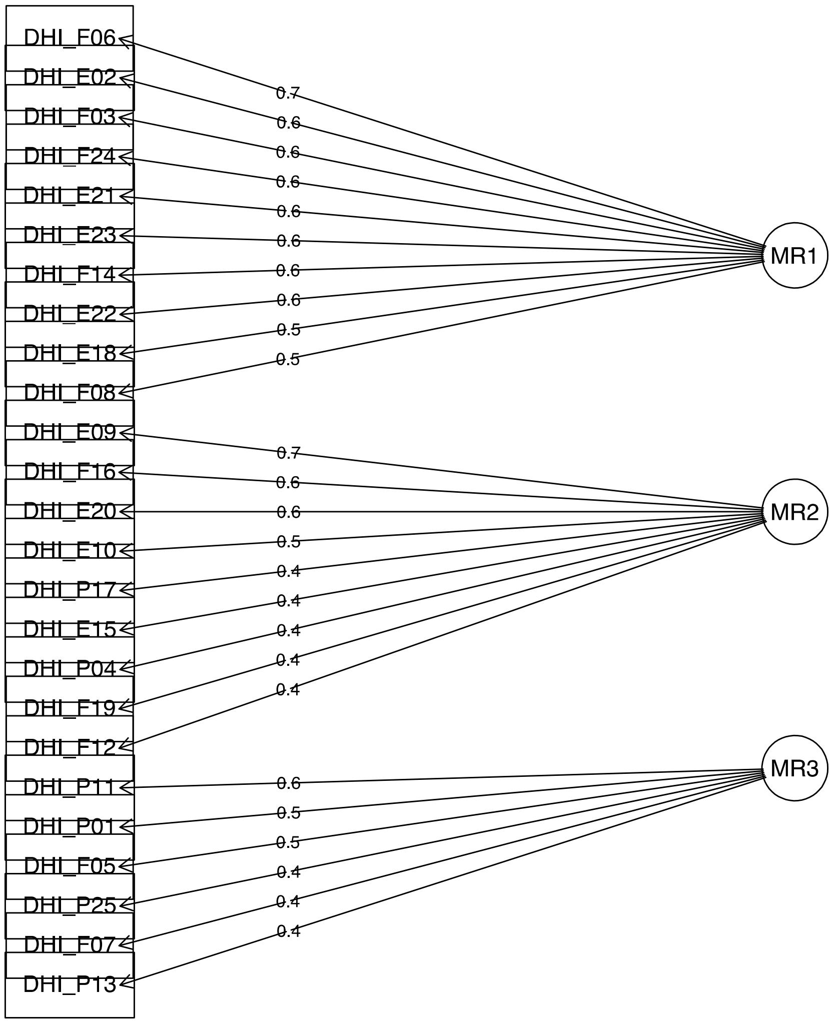

A three-factor structure (minimum residual fitting method) was found, differing from the distribution of items into the three canonical E, F, and P components. The arrows represent best item-factor association, and the numbers represent loadings.

Confirmatory Factor Analysis (CFA) on DHI

The Confirmatory Factor Analysis of DHI data rejects the factorization based on the canonical components E, F, and P: Tucker-Lewis index 0.736 (TLI ranges between 0 and 1, good fit is indicated by TLI > 0.95).

Estimator	ML
Optimization method	NLMINB
Number of model parameters	53

	Used	Total
Number of observations	272	277

Model Test User Model:

Test statistic	824.820
Degrees of freedom	272
P-value (Chi-square)	0.000

Model Test Baseline Model:

Test statistic	2608.994
Degrees of freedom	300
P-value	0.000

User Model versus Baseline Model:

Comparative Fit Index (CFI)	0.761
Tucker-Lewis Index (TLI)	0.736

Loglikelihood and Information Criteria:

Loglikelihood user model (H0)	-11375.798
Loglikelihood unrestricted model (H1)	-10963.388
Akaike (AIC)	22857.596
Bayesian (BIC)	23048.704
Sample-size adjusted Bayesian (BIC)	22880.655

Root Mean Square Error of Approximation:

RMSEA	0.086
90 Percent confidence interval - lower	0.080
90 Percent confidence interval - upper	0.093
P-value RMSEA <= 0.05	0.000

Standardized Root Mean Square Residual:

SRMR	0.076

Parameter Estimates:

Standard errors	Standard
Information	Expected
Information saturated (h1) model	Structured

Analysis of SVQ scores

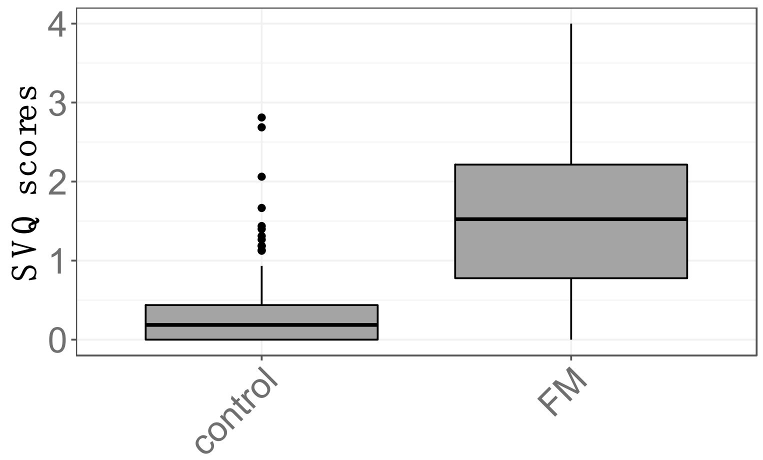

Comparison of SVQ scores between FM and controls

	Min.	1st Qu.	Median	Mean	3rd Qu.	Max.	NA's
FM	0.0000	0.7778	1.5263	1.5316	2.2164	4.0000	10
controls	0.0000	0.0000	0.1875	0.4076	0.4375	2.8125	0

Wilcoxon rank sum test with continuity correction
W = 18383, p-value < 2.2e-16
alternative hypothesis: true location shift is not equal to 0

Correlations between SVQ scores and painDETECT and Pain Intensity scores (Spearman's rank correlation coefficient).
PainDETECT: 0.3488208
Pain Intensity: 0.2528989

Correlations between SVQ scores and the scores of each pain type (Spearman's rank correlation coefficient).
Pressure pain: 0.2166736
Occasional pain: 0.3020789
Burning: 0.2440982
Numbness: 0.2845587
Sudden pain: 0.2355161
Tingling: 0.3194206
Light contact: 0.2985977

Correlations between SVQ scores and the scores assigned by patients to their childhood and youth periods (Spearman's rank correlation coefficient).
Childhood: -0.226222
Youth: -0.2479047

Mean SVQ scores in patients subdivided according to the source of FM diagnosis.

Other	Neurologist	Rheumatologist
1.514156	1.598081	1.536110

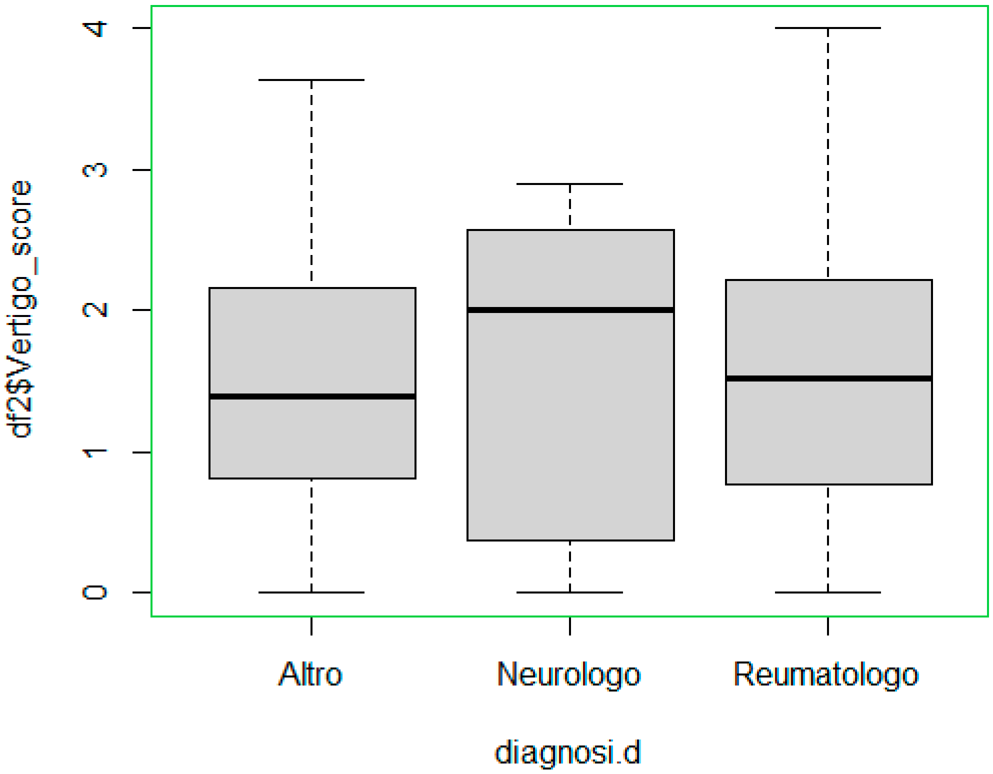

Boxplot charts of SVQ scores in patients subdivided according to the source of FM diagnosis. Left: other; middle: neurologist; right: rheumatologist.

Comparisons between the distributions of SVQ scores classified by the absence/presence (FALSE/TRUE) of the psychological condition of each item of the CBA-H test (Wilcoxon rank sum test with continuity correction).

Anxiety

W = 9463, p-value = 0.3811

$\`FALSE\`

Min.	1st Qu.	Median	Mean	3rd Qu.	Max.	NA's
0.0000	0.8065	1.4737	1.4755	2.1053	4.0000	6

$\`TRUE\`

Min.	1st Qu.	Median	Mean	3rd Qu.	Max.	NA's
0.0000	0.7708	1.5298	1.5889	2.3735	3.7857	4

FALSE	TRUE
141	136

Health-care related fears

```
W = 6464.5, p-value = 0.000873
$`FALSE`
   Min. 1st Qu.  Median    Mean 3rd Qu.    Max.    NA's
 0.0000  0.6667  1.3529  1.4454  2.1151  4.0000       9
$`TRUE`
   Min. 1st Qu.  Median    Mean 3rd Qu.    Max.    NA's
  0.000   1.418   2.029   1.968   2.564   3.786       1
FALSE  TRUE
  232    45
```

Depression

```
W = 9726.5, p-value = 0.002407
$`FALSE`
   Min. 1st Qu.  Median    Mean 3rd Qu.    Max.    NA's
 0.0000  0.6316  1.3709  1.4012  2.0573  4.0000      10
$`TRUE`
   Min. 1st Qu.  Median    Mean 3rd Qu.    Max.
  0.000   1.056   1.667   1.792   2.474   3.786
FALSE  TRUE
  188    89
```

Emotional instability-Depressive mood

```
W = 7327, p-value = 0.0001119
$`FALSE`
   Min. 1st Qu.  Median    Mean 3rd Qu.    Max.    NA's
 0.0000  0.2755  0.8586  1.0672  1.7982  3.1667       4
$`TRUE`
   Min. 1st Qu.  Median    Mean 3rd Qu.    Max.    NA's
 0.0000  0.9412  1.6316  1.6385  2.3158  4.0000       6
FALSE  TRUE
   54   223
```

Psychophysical wellbeing (TRUE = absence of condition)

```
W = 1617.5, p-value = 0.4046
$`FALSE`
   Min. 1st Qu.  Median    Mean 3rd Qu.    Max.    NA's
 0.1053  0.6053  1.3889  1.2698  1.9474  2.3158       1
$`TRUE`
   Min. 1st Qu.  Median    Mean 3rd Qu.    Max.    NA's
 0.0000  0.7778  1.5263  1.5428  2.2333  4.0000       9
FALSE  TRUE
   12   265
```

Perceived psychophysical stress

```
W = 8849.5, p-value = 0.005197
$`FALSE`
   Min. 1st Qu.  Median    Mean 3rd Qu.    Max.    NA's
```

```
 0.0000  0.6667  1.4000  1.4253  2.0690  3.6429       7
$`TRUE`
   Min. 1st Qu.  Median    Mean 3rd Qu.    Max.    NA's
  0.000   1.029   1.847   1.799   2.527   4.000       3
FALSE  TRUE
  198    79
```

Neuroticism

```
W = 8598, p-value = 0.5613
$`FALSE`
   Min. 1st Qu.  Median    Mean 3rd Qu.    Max.    NA's
 0.0000  0.6842  1.5707  1.4972  2.1812  3.7857       6
$`TRUE`
   Min. 1st Qu.  Median    Mean 3rd Qu.    Max.    NA's
 0.0000  0.8421  1.5000  1.5917  2.3684  4.0000       4
FALSE  TRUE
  176   101
```

Introversion/extroversion (TRUE = tendency to introversion)

```
W = 7277, p-value = 0.06352
$`FALSE`
   Min. 1st Qu.  Median    Mean 3rd Qu.    Max.    NA's
 0.0000  0.9605  1.6491  1.6609  2.3083  3.7857       2
$`TRUE`
   Min. 1st Qu.  Median    Mean 3rd Qu.    Max.    NA's
 0.0000  0.6316  1.3889  1.4516  2.2105  4.0000       8
FALSE  TRUE
  104   173
```

Social anxiety

```
W = 9458, p-value = 0.01233
$`FALSE`
   Min. 1st Qu.  Median    Mean 3rd Qu.    Max.    NA's
 0.0000  0.6429  1.3333  1.4282  2.0588  4.0000       7
$`TRUE`
   Min. 1st Qu.  Median    Mean 3rd Qu.    Max.    NA's
 0.0000  0.9474  1.6491  1.7348  2.4088  3.7368       3
FALSE  TRUE
  184    93
```

Haste and Impatience

```
W = 10580, p-value = 0.007315
$`FALSE`
   Min. 1st Qu.  Median    Mean 3rd Qu.    Max.    NA's
 0.0000  0.5816  1.3431  1.3872  2.0000  4.0000       6
$`TRUE`
   Min. 1st Qu.  Median    Mean 3rd Qu.    Max.    NA's
 0.0000  0.9722  1.6842  1.6907  2.3944  3.7857       4
```

```
FALSE  TRUE
  146   131
```

Excessive Involvement

```
W = 8855.5, p-value = 0.9759
$`FALSE`
   Min. 1st Qu.  Median    Mean 3rd Qu.    Max.    NA's
  0.000   0.750   1.579   1.541   2.316   4.000       6
$`TRUE`
   Min. 1st Qu.  Median    Mean 3rd Qu.    Max.    NA's
 0.0000  0.7807  1.5132  1.5230  2.2079  3.7857       4
FALSE  TRUE
  131   146
```

Hostility

```
W = 1310, p-value = 0.04751
$`FALSE`
   Min. 1st Qu.  Median    Mean 3rd Qu.    Max.    NA's
  0.000   0.739   1.500   1.510   2.211   4.000      10
$`TRUE`
   Min. 1st Qu.  Median    Mean 3rd Qu.    Max.
  1.250   1.531   2.158   2.323   3.026   3.737
FALSE  TRUE
  270     7
```

Inability to Relax

```
W = 6031, p-value = 5.072e-05
$`FALSE`
   Min. 1st Qu.  Median    Mean 3rd Qu.    Max.    NA's
  0.000   1.053   1.892   1.832   2.567   3.786       1
$`TRUE`
   Min. 1st Qu.  Median    Mean 3rd Qu.    Max.    NA's
 0.0000  0.5263  1.3333  1.3340  2.0000  4.0000       9
FALSE  TRUE
  107   170
```

Interpersonal Difficulties

```
W = 10832, p-value = 6.266e-05
$`FALSE`
   Min. 1st Qu.  Median    Mean 3rd Qu.    Max.    NA's
 0.0000  0.5447  1.2565  1.3429  2.0000  4.0000       8
$`TRUE`
   Min. 1st Qu.  Median    Mean 3rd Qu.    Max.    NA's
  0.000   1.222   1.889   1.842   2.529   3.786       2
FALSE  TRUE
  174   103
```

Leadership/Competitiveness

```
W = 7458, p-value = 0.1443
$`FALSE`
   Min. 1st Qu.  Median    Mean 3rd Qu.    Max.    NA's 
 0.0000  0.8158  1.6316  1.5980  2.3158  4.0000       6 
$`TRUE`
   Min. 1st Qu.  Median    Mean 3rd Qu.    Max.    NA's 
 0.0000  0.6798  1.2786  1.4205  2.0167  3.4706       4 
FALSE  TRUE 
  173   104 
```

Irritability

```
W = 7033.5, p-value = 0.4961
$`FALSE`
   Min. 1st Qu.  Median    Mean 3rd Qu.    Max.    NA's 
 0.0000  0.8586  1.5333  1.5860  2.3842  3.4706       2 
$`TRUE`
   Min. 1st Qu.  Median    Mean 3rd Qu.    Max.    NA's 
 0.0000  0.7005  1.4721  1.5087  2.1684  4.0000       8 
FALSE  TRUE 
   81   196 
```

Comparisons between the distributions of SVQ scores classified by the absence/presence (0/1) of each symptom other than pain (Wilcoxon rank sum test with continuity correction).

Brain fog

```
W = 3781, p-value = 0.001599
$`0`
   Min. 1st Qu.  Median    Mean 3rd Qu.    Max.    NA's 
 0.0000  0.3529  1.1333  1.1619  2.0000  4.0000       3 
$`1`
   Min. 1st Qu.  Median    Mean 3rd Qu.    Max.    NA's 
 0.0000  0.8824  1.6316  1.6201  2.3158  3.7857       6 
  0   1 
 52 223 
```

Dizziness

```
W = 5271, p-value = 2.737e-06
$`0`
   Min. 1st Qu.  Median    Mean 3rd Qu.    Max.    NA's 
 0.0000  0.4211  0.9737  1.1713  1.9737  3.4706       6 
$`1`
   Min. 1st Qu.  Median    Mean 3rd Qu.    Max.    NA's 
  0.000   1.000   1.684   1.735   2.357   4.000       3 
  0   1 
100 175 
```

Migraine

```
W = 5601, p-value = 0.0001215
```

```
$`0`
   Min. 1st Qu.  Median    Mean 3rd Qu.    Max.    NA's
 0.0000  0.4444  1.1111  1.2104  1.8421  3.4706       5
$`1`
   Min. 1st Qu.  Median    Mean 3rd Qu.    Max.    NA's
 0.0000  0.9474  1.6316  1.6993  2.3529  4.0000       4
  0   1
 94 181
```

Anxiety

```
W = 6906, p-value = 0.02184
$`0`
   Min. 1st Qu.  Median    Mean 3rd Qu.    Max.    NA's
 0.0000  0.4926  1.2917  1.3552  2.0263  3.4706       1
$`1`
   Min. 1st Qu.  Median    Mean 3rd Qu.    Max.    NA's
 0.0000  0.8896  1.5941  1.6445  2.3480  4.0000       8
 0   1
101 174
```

Photophobia

```
W = 6616, p-value = 0.0003992
$`0`
   Min. 1st Qu.  Median    Mean 3rd Qu.    Max.    NA's
  0.000   0.600   1.263   1.314   2.000   4.000       5
$`1`
   Min. 1st Qu.  Median    Mean 3rd Qu.    Max.    NA's
 0   1
134 141
```

Depression

```
W = 7409.5, p-value = 0.08913
   Min. 1st Qu.  Median    Mean 3rd Qu.    Max.    NA's
 0.0000  0.6667  1.4737  1.4517  2.1579  4.0000       5
$`1`
   Min. 1st Qu.  Median    Mean 3rd Qu.    Max.    NA's
 0.0000  0.9474  1.6316  1.6646  2.3333  3.7857       4
 0   1
166 109
```

Nausea

```
W = 6219, p-value = 0.0005198
$`0`
   Min. 1st Qu.  Median    Mean 3rd Qu.    Max.    NA's
  0.000   0.600   1.294   1.367   2.053   4.000       6
$`1`
   Min. 1st Qu.  Median    Mean 3rd Qu.    Max.    NA's
  0.000   1.000   1.778   1.811   2.579   3.786       3
```

```
 0   1 
171 104 

Diplopia
W = 5495, p-value = 3.401e-05
$`0`
   Min. 1st Qu.  Median    Mean 3rd Qu.    Max.    NA's 
 0.0000  0.6213  1.2500  1.3619  2.0263  4.0000       8 
$`1`
   Min. 1st Qu.  Median    Mean 3rd Qu.    Max.    NA's 
  0.000   1.263   1.889   1.870   2.502   3.786       1 
 0   1 
183  92 

Gut disorders
W = 3297, p-value = 0.186
$`0`
   Min. 1st Qu.  Median    Mean 3rd Qu.    Max.    NA's 
 0.0000  0.7059  1.4737  1.5070  2.2000  4.0000       9 
$`1`
   Min. 1st Qu.  Median    Mean 3rd Qu.    Max. 
 0.1053  0.9412  1.6842  1.7383  2.4737  3.3636 
  0   1 
242  33 

Tinnitus
W = 2346, p-value = 0.2468
$`0`
   Min. 1st Qu.  Median    Mean 3rd Qu.    Max.    NA's 
 0.0000  0.8355  1.5479  1.5526  2.2193  4.0000       9 
$`1`
   Min. 1st Qu.  Median    Mean 3rd Qu.    Max. 
 0.0000  0.4641  0.8235  1.2721  2.1184  3.3158 
 0   1 
259  16 

Fatigue
W = 293, p-value = 0.4408
$`0`
   Min. 1st Qu.  Median    Mean 3rd Qu.    Max.    NA's 
 0.1765  0.4216  0.6667  1.0810  1.5333  2.4000       1 
$`1`
   Min. 1st Qu.  Median    Mean 3rd Qu.    Max.    NA's 
 0.0000  0.7865  1.5263  1.5367  2.2134  4.0000       9 
  0  1 
  4 273 

Sleep disturbance
```

```
W = 2982.5, p-value = 0.4993
$`0`
   Min. 1st Qu.  Median    Mean 3rd Qu.    Max.    NA's
 0.0000  0.7706  1.3158  1.3888  2.2053  3.0000       1
$`1`
   Min. 1st Qu.  Median    Mean 3rd Qu.    Max.    NA's
 0.0000  0.7778  1.5479  1.5476  2.2222  4.0000       9
 0   1
 28 249
```

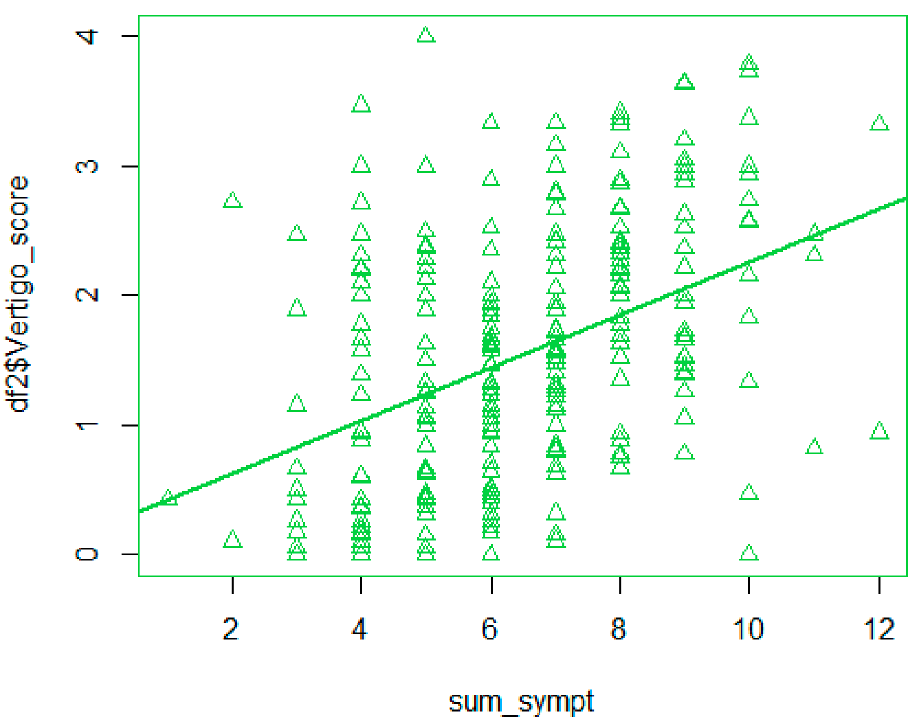

Plot of SVQ scores vs. the number of reported symptoms other than pain.

Exploratory Factor Analysis of the DHI and SVQ items

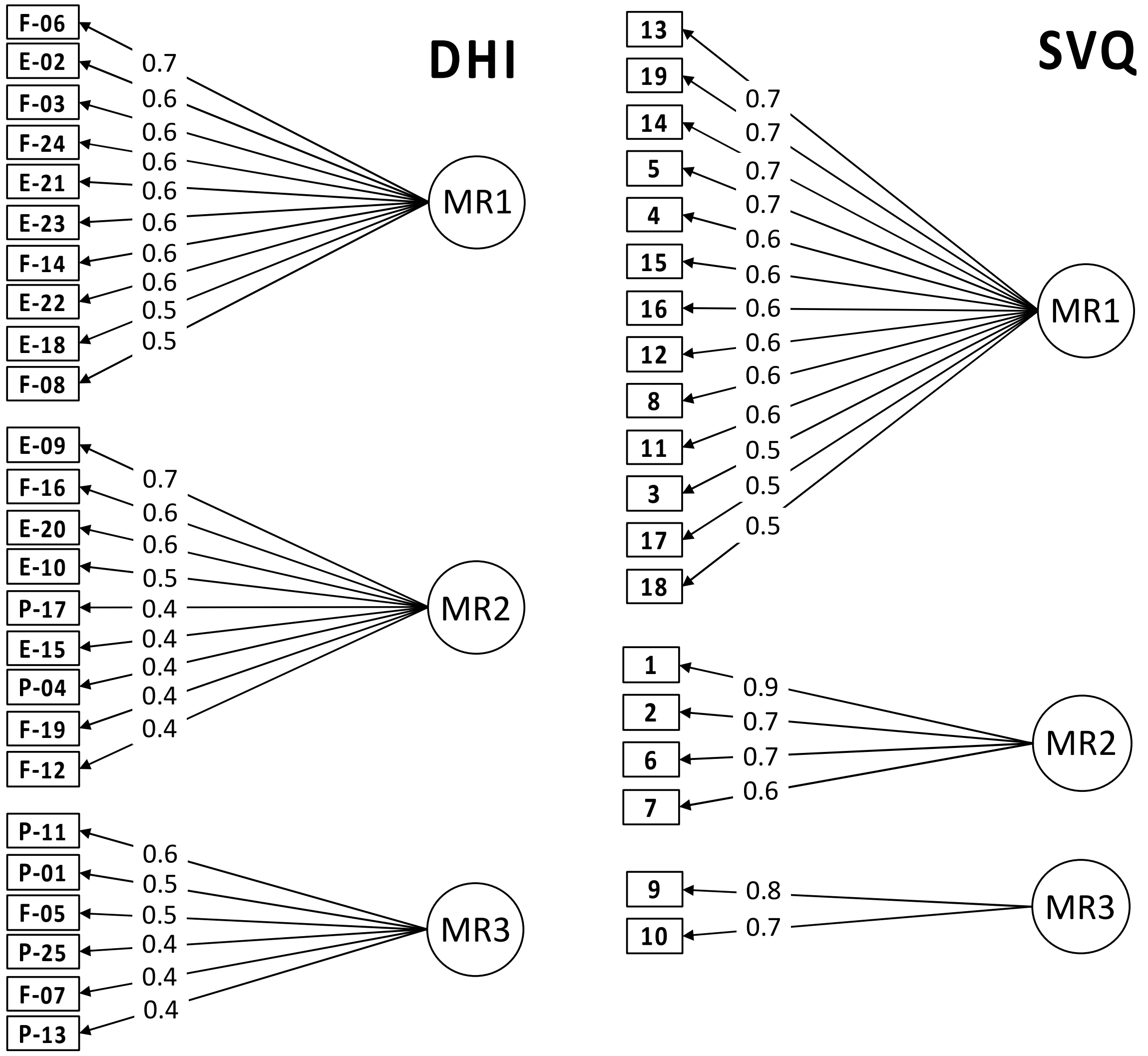

The arrows represent the best item-factor association, and the numbers represent the loadings.

The Confirmatory Factor Analysis of DHI data rejects the factorization based on the canonical test components E, F, and P, Tucker-Lewis Index (TLI) = 0.736 (TLI ranges between 0 and 1, good fit is indicated by TLI > 0.95).
